# A Keystone-Taxa SynCom Reveals *Chlorella*–Microbiome–Plant Communication and Enhances Suppression of *Fusarium oxysporum*

**DOI:** 10.4014/jmb.2601.01023

**Published:** 2026-04-03

**Authors:** Hwa-Jung Lee, Jin Hwan Lee, Youn-Sig Kwak

**Affiliations:** 1 Institute of Agriculture and Life Science, Gyeongsang National University, Jinju 52828, Republic of Korea; 2 Department of Smart Green Resources, Dong-A University, Busan 49315, Republic of Korea; 3 Department of Plant Medicine and, Research Institute of Life Science, Gyeongsang National University, Jinju 52828, Republic of Korea

**Keywords:** *Chlorella fusca*, Keystone taxa, Synthetic community, Microbiome community, *Fusarium oxysporum*

## Abstract

*Chlorella*, a microalga renowned for its high protein and lipid content, is extensively used as a biofertilizer due to its plant growth promotion and disease suppression capabilities. To reveal, *Chlorella*-microbiome-plant communication, in-depth microbiota structure and network analyses were conducted. As a result, keystone taxa, which are microbial element in interaction with *Chlorella*, keystone taxa (*Psedomonas*, *Duganella*, *Brevibacterium*) interaction with *C. fusca* CHK0059 within plant hosts were identified. Here, however, the mechanistic insights into these interactions remain limited. Therefore, we aimed to investigate the characteristics of keystone taxa to elucidate *Chlorella* effects on plants. By applying various substances, including *Chlorella*, *Chlorella* methanol extract (methanol extract), and D-mannitol, we observed changes in microbial distribution and diversity, with a notable increase in *Pseudomonas* abundance following 2% D-mannitol treatment. Additionally, we assessed the impact of *Chlorella* on plant growth and disease suppression, finding that a synthetic community (SynCom) of keystone taxa exhibited enhanced antifungal effects against *Fusarium oxysporum* in both strawberry and tomato, compared to individual strains. The findings in this study suggested the fundamental data that the SynCom can contribute to the mechanism of action of *C. fusca* CHK0059 and expect to maximize the effect of *Chlorella* when combined.

## Introduction

Microalgae, well known as a biofertilizers, are the microscopic eukaryotic organism that can release oxygen through photosynthesis and produce biomass for feed, useful compounds, and fuel [1, 2]. They use sunlight/light water and carbon dioxide to accumulate biomass via a photosynthetic process [3]. Microalgal biomass can be employed as an organic slow-release fertilizers and are studied largely due to its commercial importance as they have high content proteins, vitamins, and fatty acids [4, 5]. Preliminary research has shown that these nutrients can promote plant growth and protect plants from external environmental factors, leading to an increase in studies utilizing microalgae [6]. Among the microalgae, *C. vulgaris*, *C. fusca*, *Acutodesmus dimorphus*, and *Spirulina platensis* have been observed to enhance plant growth and yield [4, 7, 8]. Of these, *Chlorella* spp. is easy to cultivate, has high productivity, and rich in nutrients, making them suitable for human consumption as well as for use in agriculture as animal feed, wastewater purification, and biofertilizer [9]. Applying *Chlorella* extract to wheat leaves has been shown to increase yield by 140%, and *Chlorella* can be used to produce fertilizers [10].

*Chlorella* biomass contained 25~30% protein, 6~10% carbohydrate, and 30~40% lipid [11]. It contains valuable proteins and lipids, including polyunsaturated fatty acids (PUFA). *Chlorella* is currently sold for its high protein content, which ranges from 11% to 58%, as well as its rich vitamin profile, including significant amounts of β-carotene, biotin, vitamin B12, and small quantities of vitamin E [12]. It also has reach in minerals and trace elements, including magnesium, iron and zinc, and manganese [13]. Algal lipids have many uses in biofuels like biodiesel, food, feedstocks, and therapeutics [14, 15]. Lipids are crucial in the metabolism and growth of microalgae, acting as a vital energy and carbon source [16]. The previous studies on lipid accumulation in freshwater *Chlorella* under salt stress have yielded valuable insights [17]. Although studies have been conducted on the effects of *Chlorella* and its effects on lipid accumulation, research on the mechanism of action of *Chlorella* remains inadequate.

Interactions between *Chlorella* and microorganisms such as bacteria can purify wastewater and positively affect plants, highlighting the importance of studying the interactions between *Chlorella* and microorganisms [18-21]. The microbial communities that exist in various plant tissues and continuously interact with plants are referred to as the ‘microbiome’. The microbiome can form complex symbiotic relationships with plants and plays a crucial role in promoting plant productivity and health in natural environments [22, 23]. Many studies have identified the existence of a 'core microbiome,' which is a subset of microbial lineages consistently associated with a given host across various environments [24]. The core microbes are known to have biological characteristics that directly or indirectly benefit plants, such as promoting plant growth through metabolic products and secretions or inhibiting plant pathogens through nutrient competition [25-27]. These microbes with such biological traits are called plant growth-promoting bacteria (PGPB). It has been found that these microbes can act as eco-friendly biological control agents to protect plants from environmental pollution rather than using chemical control methods, leading to ongoing research into control methods using PGPB [28]. *Bacillus subtilis*, a well-known PGPB, is famous for its plant growth-promoting effects and has also been shown to inhibit plant diseases through the production and release of specific bioactive compounds [29, 30]. Additionally, *Pseudomonas fluorescens*, another well-known PGPB, has shown plant growth promotion (PGP) abilities as a phytostimulator, nitrogen fixation capability, and enhancement of soil health and plant biomass [31, 32]. Application of *Chlorella* to plants enhances plant growth and disease suppression through interactions with the microbial communities present in various parts of the plant. *C. fusca* CHK0059 is already known to show the increase on the growth and quality of Chinese chives and suppress anthracnose caused by *C. orbiculare* [8, 33]. Treatment of *C. fusca* CHK0059 on strawberries showed an inhibitory effect on *Fusarium oxysporum*, the causal pathogen of Fusarium wilt disease [34].

*C. fusca* CHK0059 was applied to the model plant *Arabidopsis thaliana* to investigate microbial community response. In network analysis, they revealed the microorganisms in the rhizosphere of *A. thaliana* that had a positive correlation with *Chlorella*. The microorganisms positively correlated with *Chlorella* in *A. thaliana* were identified as *Pseudomonas* and *Brevibacterium* [35]. Experiments were conducted to identify the microbial communities interacting with *Chlorella* in strawberry tissues at different growth stages. Microbial community analysis revealed a complex distribution of microorganisms positively correlated with *Chlorella* in the rhizosphere of strawberries [36]. Additionally, when *C. fusca* CHK0059 was applied to strawberries during the seedling stage, an increase in chlorophyll content was observed in the CHK0059 treatment. During the strawberry cultivation time, *Chlorella* increased in leaf number and shoot length was observed in strawberry. Microbial community analysis showed increased microbial diversity in the roots with CHK0059 treatment, but the keystone taxa, the microorganism elements influencing the plant, were positively correlated with *Chlorella* in the rhizosphere. Network analysis identified *Pseudomonas*, *Duganella*, and *Rhizomicrobium* as the keystone taxa in the rhizosphere positively correlated with CHK0059 treatment [37].

Synthetic microbial community (SynCom) approach is emerging research that incorporates a synthetic biology approach with microbial community analysis, metagenomic, and bioinformatic approaches [38]. SynCom are constructed by incorporating multiple taxa under well-defined conditions to mimic the function of a microbiome [38]. SynCom can exclude variable environmental influences and reduce the damage of abiotic and biotic stress. The basis of SynCom is collecting information on the overall plant microbiome composition required to form a “keystone microbiome” [39]. SynCom can boost the plant immune system against plant disease pathogen. Several studies have been reported that SynCom can induce disease resistance and promote plant growth against the pathogen infection [40, 41]. For example, pepper and tomato have highlighted positive impacts of PGPR on rhizosphere microbiome assembly and richness indices [42]. The development and application of SynCom can be a sustainable approach to increase plant growth and plant tolerance against plant disease.

Certain microorganisms were identified as interacting with *Chlorella*, yet their positive impact on plants has not been conclusively demonstrated [37]. This study seeks to detail the responses of microorganisms, such as *Pseudomonas*, *Duganella*, and *Brevibacterium*, to various *Chlorella*-derived substances, including *Chlorella* itself, its methanol extract, and D-mannitol, within the strawberry rhizosphere. By observing changes in microbial community structure and evaluating the synergistic effects of these keystone taxa when co-applied with *Chlorella*, we aim to elucidate their biological characteristics that contribute to plant growth promotion and disease suppression. Furthermore, we assess the efficacy of these interactions as a SynCom in controlling *F. oxysporum* in both strawberries and tomatoes. This comprehensive approach provides fundamental insights into the potential of these keystone taxa as a novel mechanism of action for *C. fusca* CHK0059, positioning them as promising candidates for biological control agents in agricultural applications.

## Materials and Methods

### Strawberry Growth Condition and Rhizosphere Microbiome Fraction

**Fig. 1:**
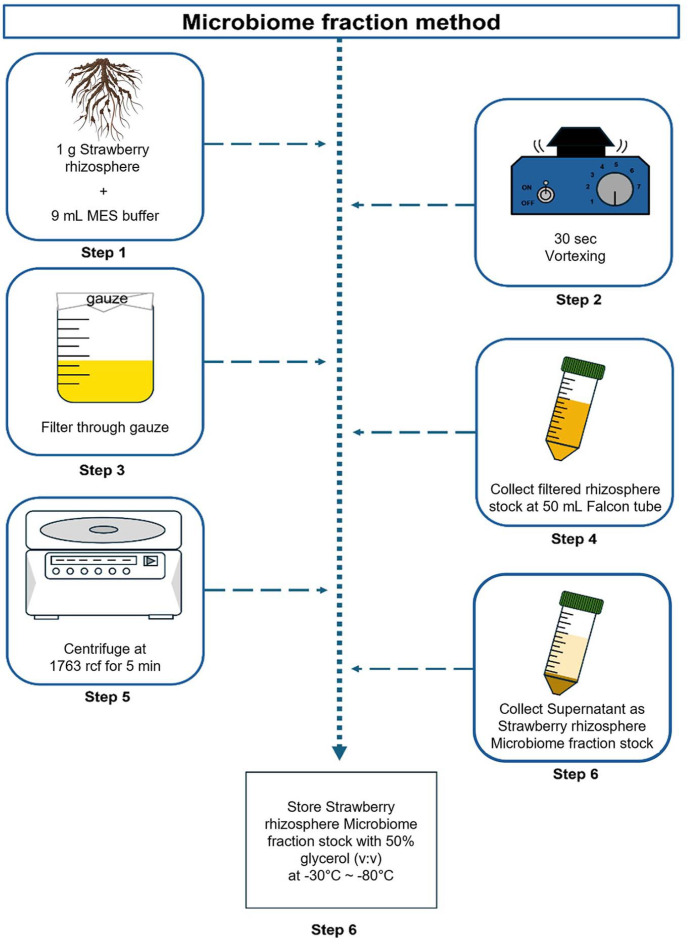
Graphical illustration about strawberry microbiome fraction stock methods. Mix 1 g of strawberry soil and 9 ml of MES buffer. The mixed stock was primary filtered using gauze. After filtering the stock, use a centrifuge to cell down the soil particles, and then sample the rhizosphere microbiome stock from supernatant which the soil microorganisms emerged. Store the strawberry rhizosphere microbiome stock with 50% glycerol (v:v) at –80^0^C.

The strawberries (cv. Seolhyang) in cultivation time used a greenhouse at Gyeongsang National University (Jinju, Republic of Korea), from mid-November 2022 to Feburary in 2023. Five strawberry plants were randomly selected, and the rhizosphere were collected by initially shaking off the soil from the strawberry plants for removing soil located farther away from the roots and then collecting the rhizosphere soil as close as possible to the strawberry roots. The strawberry microbiome fraction stocks (MF) were produced using the below protocol (Fig. 1). The MF was extracted from ‘Seolhyang’ strawberries rhizosphere. 1 g of the collected rhizosphere from strawberries and 9 ml MES buffer (97.63 g of MES free acid, total volume per L, pH 5.7) mixed and vortexed for 30 sec. Then the soil stocks were filtered through Mira cloth into a 50 ml tube to remove the soil particles. The filtered soil stock tubes were centrifuged in 1763 ×*g* for 5 min. The collected supernatant from each tube after centrifuged. Then added 50% glycerol in a 1:1 ratio (v/v) and collected in a 5 ml tubes. The produced MF stocks were stored at -80^0^C.

### *Chlorella* Culture Condition

*C. fusca* CHK0059 culture stock was used in this study. The *Chlorella fusca* strain CHK0059 was provided by the CHK0059 culture stock in the medium made from the F&B Nature Co. Ltd., (Republic of Korea). *C. fusca* CHK0059 were streaked on R2A medium (MB cell R2A 3.15 g, Agar 20 g per L) to collect single colony of *C. fusca* CHK0059 and cultured as follows: inoculate the single fresh CHK0059 colony to R2A broth (MB cell R2A 3.15 g per L), temperature; 28°C, period; 5 days, light; 16 h and dark; 8 hr in a day.

### *C. fusca* CHK0059 Culture Media Extracted by Methanol

CHK0059 and the most abundant components containing CHK0059 culture medium extracted with methanol (methanol extract) were treated to strawberry rhizosphere microbiome fraction. Preliminary experiments showed that the CHK0059 keystone taxa were involved in the hexitol fermentation pathway and were treated with mannitol, which can promote the pathway, to determine how the plant's microorganisms interact with each substance. To check whether it would react, the methanol extract was extracted from the CHK0059 sludge using methanol, which has a high solved polarity. Additionally, in methanol extract of the total phenolic content was analysed to be 0.835 GAE mg/g, and the total flavonoid content was analysed to be 1.89 QE mg/g, confirming that it has functional ingredients. Methanol extract is extracted at room temperature by adding the same volume of methanol to CHK0059 sludge and left overnight for 7 days for extraction, performing evaporation using an evaporator (EYELA Oil Bath model OSB-2000, Tokyo Rikakika Co., Ltd., Japan), and then using the evaporator along with methanol extract (710 mg) and 2 g of silica gel (70-230 mesh). It was produced through purification. Methanol extract was used at dilutions of 1/10 and 1/100 of the original concentration (methanol extract 0.1x, methanol extract 0.01x).

### Strawberry Rhizosphere MF Stock Treated CHK0059

In order to investigate the interaction between CHK0059 and microorganisms distributed in the rhizosphere, where numerous microorganisms interacting with CHK0059 and its constituents are known to exist, a study was conducted to observe the diversity changes of microorganisms in strawberry rhizosphere upon treatment with CHK0059 and CHK0059 cultivation broth methanol extracts, as well as D-mannitol, which could potentially influence the "hexitol fermentation to lactate, formate, ethanol and acetate" pathway of CHK0059 keystone taxa [37]. First, to ascertain the immediate effects of *C. fusca* CHK0059 and its constituents on the survival of microorganisms present in the strawberry rhizosphere fraction, the extracted strawberry rhizosphere MF stock was aliquoted about 3 ml each into 5 ml tubes. CHK0059-related substances (CHK0059, methanol extract, and D-mannitol) were treated at a ratio of 1/10 of the total volume of each treatment group.

To determine the concentration that affects microbial diversity, CHK0059-related substances (CHK0059, methanol extract, D-mannitol) were treated by dividing them by concentration (CHK0059 10^7^ cell/ml, CHK0059 10^6^ cell/ml, methanol extract 0.1Χ, methanol extract 0.01Χ, D-mannitol 2%, D-mannitol 0.2%). After treating, the samples were cultured in a 27°C shaking incubator for designated time intervals (12 h, 24 h, 36 h, and 48 h), and microbial viability was assessed by dilution plating on R2A media, followed by incubation at 27°C for 5 days to quantify the microbial density of untreated control groups and CHK0059 substance-treated groups (Sterilized Chlorella, Chlorella 10^7^, Chlorella 10^6^, Methanol extract 0.1x, Methanol extract 0.01x, Mannitol 2%, Mannitol 0.2%).

### Microbiome Analysis of Strawberry Rhizosphere MF

To assess changes in the diversity of microorganisms present within the MF stocks treated with various substances, DNA was extracted from each treatment group. DNA extraction was performed using the FastDNA SPIN Kit for Soil (MP Biomedicals, USA) to extract DNA from microorganisms present in each sample. Each sample, contained in a Lyzing Matrix E tube, was pipetted with 1 ml and supplemented with 978 μl of sodium phosphate buffer and 122 μl of MT buffer. The Lyzing Matrix E tubes containing the samples were homogenized using a FastPrep instrument at 6.0 m/s for 40 sec. The homogenized Lyzing Matrix E tubes were then centrifuged at 12,470 ×*g* for approximately 5 min. The supernatant was transferred to a new 1.7 ml E-tube, and 250 μl of PPS was added, followed by several inversions. After inversion, centrifugation was repeated under the same conditions. Following centrifugation, the supernatant was transferred to a 5 ml tube, and 1 ml of Binding Matrix solution was added. The mixture was inverted for 2 min and left at room temperature for 3 min to allow for phase separation. After phase separation, 500 μl of the supernatant was removed. The remaining solution was transferred to a SPIN Filter tube and centrifuged at 12,470 ×*g* for 1 min to remove the lower phase. The remaining sample was transferred to the SPIN Filter tube and centrifuged again under the same conditions. After centrifugation, the lower phase was removed, and 500 μl of SEWS-M solution was added to the SPIN Filter tube. Inversion was performed five times for washing. Following washing, centrifugation was conducted at 12,470 ×*g* for 1 min, and the filter was attached to a new catch tube. The filter was then air-dried at room temperature for approximately 5 min. After air-drying at room temperature, 50 μl of DES buffer was added and allowed to react for 5 min. The mixture was then centrifuged at 12,470 ×*g* for 3 min to complete DNA extraction. The extracted DNA was then assessed for quality using a NanoDrop 2000C (ThermoFisher, USA).

PCR was conducted using the 515F (5′-TCGTCGGCAGCGTCAGATGTGTATAAGAGACAGGTGYCAGCMGCCGCGGTAA) and 805R (5′-GTCTCGTGGGCTCGGAGATGTGTATAAGAGACAGGACTACHVGGGTATCTAATCC) primers. PCR reactions were performed with 100 ng of DNA, 10 pmol primers, and 13.5 μl of KAPA HiFi HotStart ReadyMix (Roche, Switzerland), with a total volume of 25 μl. The amplification conditions consisted of an initial denaturation at 95°C for 3 min followed by 30 cycles of denaturation at 95°C for 30 sec, annealing at 55°C for 30 sec, and extension at 72°C for 30 sec. A final extension step was performed at 72°C for 5 min. The PCR products were purified using the ExpinTM Gel SV kit (Gene-all, Republic of Korea).

The purified DNA was subjected to Illumina MiSeq sequencing using the Herculase II Fusion DNA Polymerase Nextera XT Index Kit V2 kit by Macrogen (Republic of Korea), as per the protocol in part #15044223 Rev. Raw data analysis was performed using the DADA2 software package (version 4.2.0). Libraries were merged with forward and reverse reads, and chimera sequences were removed, following the reference provided in the official protocol. Furthermore, taxonomic assignment of Amplicon Sequence Variants (ASVs) was conducted using SILVA (https://www.arb-silva.de/download/arb-files/) and IDTAXA (Murali *et al*., 2018).

Alpha diversity analysis, including richness and evenness, was carried out using ggiNEXT (version 3.0.0) and phyloseq (version 1.44.0). To compare the differences in microbial community diversity each treatment group over time (12 h and 36 h), t-test was used to verify the significance [43]. For beta diversity analysis, the distribution of the top 10 ASVs in each sample was examined to represent microbial diversity between samples. Principal Coordinates Analysis (PCoA) was performed using Bray-Curtis dissimilarity metrics, and the distances between samples were statistically analysed using the PERMANOVA package through adonis analysis to determine significant differences in microbial diversity between samples, based on *p*-values [44]. Additionally, the results of alpha-diversity, beta-diversity, and PCoA were visualized using R packages.

Additionally, based on previous research indicating interactions with CHK0059, keystone taxa were identified to determine their response to CHK0059, methanol extract, and D-mannitol treatments. The relative abundance of ASVs of keystone taxa present in each treatment sample was examined to assess the density of Keystone taxa influenced by the substance treatment. The relative abundance of specific microorganisms in a sample was measured based on the abundance of ASVs present in the sample. The relative abundance of ASVs present in the sample was determined based on the 16S rRNA sequences of *Pseudomonas* spp., *Duganella* sp., and *Brevibacterium* sp., which were identified as keystone taxa in each sample. The relative abundance differences of keystone taxa in the treatment groups were analyzed for significance between the treatment groups using the nonparametric test method, the Kruskal-Wallis Rank Sum Test [45]. The results indicating significance with a p value of less than 0.05 were used as a post-hoc analysis using the Conover Test [46] to indicate significance by group.

### Plant Growth-Promoting Activity Test of Keystone Taxa

Before treating keystone taxa in the form of a Synthetic community (SynCom) on plants, their direct plant growth-promoting (PGP) activities were evaluated. *Pseudomonas* spp. (8C3D12, 6C7F4) used in this study were isolated and distributed *Pseudoduganella rivuli* FT92W (FT92W) from Korean Agricultural Culture Collection (KACC, Jeonju, Korea) and *Brevibacterium renqingii* REN4 (REN4) Korean Collection for Type Cultures (KCTC, Daejeon, Korea). The strain information is listed in Table 1. The PGP assays included protease, phosphate solubilization, nitrogen fixation, siderophore production, cellulase activity, chitinase activity, and Indole-3-acetic acid (IAA) production tests. To conduct the assays, keystone taxa strains were cultured in R2A broth media to reach an OD_600_ of 0.6, ensuring uniform stock concentrations. For the protease assay, skimmed milk agar media (20 g skim milk per L) was prepared and inoculated with 20 μl of each keystone taxa culture on paper discs (8 mm in diameter) placed at the center of the plates. After incubating at 28°C for 3 days, the length of the clear zone around each disc, indicating protease activity, was measured.

For the phosphate solubilization assay, Pikovskaya media was prepared with the following composition: yeast extract 0.5 g, dextrose 10 g, Ca_3_(PO_4_)2 5 g, (NH_4_)_2_SO_4_ 0.5 g, KCl 0.2 g, MgSO_4_ 0.1 g, MnSO_4_ 0.0001 g, FeSO_4_ 0.0001 g, and agar 15 g per L, pH adjusted to 6.8-7.0. The media were then autoclaved at 121°C for 15 min. After solidification, 20 μl of each keystone taxa culture was inoculated onto paper discs (8 mm in diameter) placed at the center of Pikovskaya agar plates [47]. Following incubation at 27°C for 5 days, the diameter of the clear zone surrounding each disc, indicative of phosphate solubilization, was measured.

To assess nitrogen fixation capability, the Keystone taxa were inoculated on Nfb Medium. The Nfb medium was prepared by adding DL-malic acid 5 g, K_2_HPO_4_ 0.6 g, MgSO_4_ 0.05 g, NaCl 0.02 g, CaCl_2_• 2H_2_O 0.02 g, micronutient solution (CuSO_4_•5H_2_O 0.4 g, ZnSO_4_•7H_2_O0.12 g, H_3_BO_3_1.4 g, Na_2_MoO_4_•2H_2_O1.0 g, MnSO_4_•H_2_O1.5 g per L) (2 ml), bromthymol blue solution (0.5% in 0.2N KOH) (2 ml), Fe(III) EDTA (1.64%) (4 ml), and vitamin solution (Biotin 10 mg, Pyridoxol HCl 20 mg per L) (1 ml) per L of water. Agar (1.75 g) was added to adjust the pH to 6.6-7.0. The medium was dispensed into test tubes (5 ml each), and then autoclaved at 121°C for 15 min to ensure sterilization. After cooling to room temperature, the sterile medium was inoculated with each keystone taxa using a loop, placing the inoculum at the center of the Nfb medium. The inoculated tubes were then incubated at 27°C for 7 days, during which the observation for a color change to blue indicated nitrogen fixation activity.

The ability to form siderophores was assessed using CAS agar medium. Before making CAS agar medium, four stock solutions (blue dye stock, minimal Media 9 salt solution stock, 20% glucose stock, NaOH stock) were prepared. First, to make the blue dye stock, three solutions were prepared. Solution 1 was made by dissolving 0.06 g of CAS (Fluka Chemicals, USA) in 50 ml of ddH_2_O. Solution 2 was made by dissolving 0.0027 g of FeCl_3_-6H_2_O in 10 ml of 10 mM HCl. Solution 3 was made by dissolving 0.073 g HDTMA in 40 ml of dH_2_O. Then, Solution 1 was mixed with 9 ml of Solution 2, and then mixed with Solution 3. This mixture was autoclaved and stored in a plastic bottle. Second, the minimal media 9 (MM9) salt solution was made by dissolving 15 g of KH_2_PO_4_, 25 g of NaCl, and 50 g of NH4Cl in 500 ml of dH_2_O. The glucose stock was made by dissolving 20 g of glucose in 100 ml of ddH_2_O. The NaOH stock was made by dissolving 25 g of NaOH in 150 ml of ddH_2_O and adjusting the final pH to 12. The casamino acid solution was made by dissolving 3 g of casamino acid in 27 ml of ddH_2_O and incubating it overnight at 4°C. A 3% of 8-hydroxyquinoline stock was prepared in chloroform, and 30 ml of this solution was mixed with the overnight-incubated in the casamino acid stock. This mixture was filtered to remove ions. After preparing all the stocks, 100 ml of MM9 solution was added to 750 ml of ddH_2_O, and 32.24 g of piperazine-N, N’-bis (2-ethanesulfonic acid) (PIPES) was dissolved. PIPES was dissolved using a magnetic bar while slowly adjusting the pH to 6.8. After adjusting the pH, 15 g of agar was added. The prepared medium was autoclaved at 121°C for 15 min. After autoclaving, the medium was cooled to 50°C, and then 30 ml of prepared casamino acid solution and 10 ml of 20% glucose solution were added. Finally, 100 ml of blue dye stock was slowly added along the wall and mixed well before pouring into plates to make the medium. An 8 mm paper disc was placed in the center of the prepared medium, and 20 μl of each bacterial spore stock (OD_600_ 0.6) was dispensed onto the disc [48]. The plates were incubated for 14 days, and the clean zone was observed.

The medium for detecting chitinase activity was prepared by dissolving 5 g of chitin, 5 g of yeast extract, 0.7 g of KH_2_PO_4_, 0.5 g of K_2_HPO_4_, 0.3 g of MgSO_4_, 0.1 g of FeSO_4_, and 0.1 g of NaCl per L, adjusting the pH to 6.5-7.0, adding 18 g of agar, and sterilizing by autoclaving. For cellulase detection, the medium was prepared by dissolving 1 g of yeast extract, 1 g of carboxymethyl cellulose (CMC), 4 g of KH_2_PO_4_, 0.05 g of MgSO_4_, 0.05 g of FeSO_4_, 2 g of CaCl_2_, and 2 g of NH_4_Cl per L, adjusting the pH to 7.0-7.4, adding 18 g of agar, and sterilizing by autoclaving. For the chitinolytic and cellulolytic reactions, an 8 mm paper disc was placed in the center of the prepared medium, and 20 μl of bacterial spore stock was dispensed onto the disc. The plates were incubated at 27°C for 5 days. After incubation, the plates were stained with 0.1% Congo red solution for 15 min and then thoroughly washed with 1 M NaCl [49].

For the IAA activity test, the Salkowski reagent method [50] was used. To prepare the Salkowski reagent, 1% L-tryptophan was dissolved in ddH_2_O and incubated overnight at 37°C with shaking, followed by 0.2 mm filtration. Tryptophan was then added to R2A medium to a final concentration of 0.1%. Keystone taxa were cultured in the R2A medium with added tryptophan and incubated at 30°C for approximately 14 days. After 14 days of incubation, the culture was centrifuged at 3,134 ×*g* for 10 min, and 1 ml of the supernatant was mixed with Salkowski reagent in a 2:1 ratio. The Salkowski reagent was prepared by mixing 35% HClO_4_ and 0.5 M FeCl_3_ in a 1:50 ratio. After mixing the supernatant and Salkowski reagent, the solution was allowed to sit for 25 min to observe a color change to pink.

### Plants (Strawberry and Tomato) Cultivation

To verify the plant growth-promoting effects and disease-suppressing effects of keystone taxa *in planta*, strawberries and tomatoes were grown for the experiment. The strawberry (*Fragaria x ananassa* Duch.) variety ‘BerriPop haruhi’ and the tomato (*Solanum lycopersicum* Mill) variety ‘Seogwang’ were selected for the experiment. After seed germination, plants with an aerial part length of about 5 cm were selected and transplanted into Boonong No. 2 pots (composed of 4% zeolite, 7% perlite, 6% vermiculite, 68% cocopeat, 14.73% peat moss, 0.201% fertilizer, 0.064% wetting agent, and 0.005% pH adjuster) with 10 plants per treatment group. One week after transplantation, microbes were applied at 10 ml (10^6^ CFU/ml) per plant for 2 weeks. Two weeks later, *F. oxysporum* was applied to induce disease, followed by additional SynCom strains applied at 10 ml per plant to ensure complete penetration of SynCom strains into the plants. SynCom strains were applied a total of three times. The plants grew under conditions of 27°C, 70% humidity, 16 h of light, and 8 h of darkness in a growth chamber.

### SynCom and *Chlorella* Cultivation and Treatment

To confirm the synergy effect of SynCom and *Chlorella* in plant systems, we prepared SynCom and CHK0059 culture solutions for plant treatment. Each strain from SynCom and CHK0059 was streaked on R2A media and cultured individually as single colonies in R2A broth at 28°C in a shaking incubator for 3 days. The cultured strain broth was centrifuged at 3,134 ×*g* for 20 min to remove the separated culture medium. After removing the medium, sterile water was added in equal volume, vortexed for 30 sec, and centrifuged again at 3,134 ×*g* for 20 min. This washing process was repeated twice to wash the culture medium. The SynCom strain spore stock, washed during the process, was prepared at a concentration of 10^6^ CFU/ml and applied to the plants with 10 ml each. CHK0059 was prepared at a concentration, at 10^7^ cells/ml, and applied at a 1/500 dilution, 10 ml per plant. For the mixed treatment of SynCom strains and CHK0059, each strain spore stock (10^6^ CFU/ml) and *Chlorella* cell stock (1/500 dilution of 10^7^ cells/ml) were mixed in a 1:1 ratio and applied at 10 ml each. To assess the disease suppression ability of CHK0059 and SynCom against *F. oxysporum* pathogen, *Fusarium* pathogen was cultured from a pre-cultured 1/5 PDA plate, inoculated into potato dextrose broth, 24 g per L (PDB), and cultured for 5 days. Spore stocks were prepared by centrifuging the microbial suspension twice.

The experimental treatments included: "Untreated," where no treatment was applied; "F," treated with *F. oxysporum* (F) only; "Treat1," treated with *Pseudomonas* sp. 8C3D12 (8C3D12) strain and F; "Treat2," treated with *Pseudoduganella rivuli* FT92W (FT92W) strain and F; "Treat3," treated with *Pseudomonas* sp. 6C7F4 (6C7F4) strain and F; "Treat4," treated with *Brevibacterium renqingii* REN4 (REN4) strain and F; "Treat5," treated with CHK0059 and F. Additionally, treatments included "Treat6," a mix of 8C3D12 and FT92W with F, confirmed keystone taxa in strawberry rhizosphere networks; "Treat7," including CHK0059 added to SynCom (8C3D12, FT92W) and F; "Treat8," all confirmed SynCom strains (8C3D12, FT92W, 6C7F4, REN4) treated with F; "Treat9," all SynCom strains with CHK0059 with F; and "Treat10," where Mannitol 2% was treated along with F, reflecting responses seen in previous studies on keystone taxa. Microbial stocks were prepared accordingly for each treatment group and experiments were conducted.

### Plant Phenotype and Disease Severity Investigation

To assess the enhanced disease suppression effect of CHK0059 following treatment with SynCom strains and *Fusarium* pathogens, Fusarium wilt symptoms were monitored in strawberries for up to 7 weeks and for 4 weeks. Phenotypic changes in strawberries and tomatoes, as well as the disease suppression capability against Fusarium wilt, were evaluated across different treatment groups. Phenotypic assessments included randomly selecting 3 plants per treatment group to measure leaf number, shoot length, shoot weight, root length, and root weight compared to untreated controls. Additionally, chlorophyll concentrations were measured to assess differences induced by SynCom treatments. Chlorophyll extraction involved cutting 3 mm^2^ leaf sections, incubating them in 0.1 g glass vials with 10 ml of 80% acetone under dark conditions at 4°C for 2 days. Chlorophyll content was then quantified using UV-spectrophotometry (NanoDrop 2000C, ThermoFisher Scientific, USA) at 645 nm and 663 nm wavelengths based on Arnon’s equation [51]. The equation is followed by C = 20.2*A_645_ + 8.02*A_663_ formula. C is the total chlorophyll contents in mg/l of acetone extract. A_645_ and A_663_are the absorptions of the extract at 645 nm and 663 nm.

To assess the disease suppression capabilities of SynCom strains, we measured the disease severity of each treatment group based on disease severity index scales (DI) for strawberry Fusarium wilt and tomato Fusarium wilt. For strawberry, the scale ranged from 0 to 5 as defined by Chae *et al*., 2015: 0 representing a healthy plant, 1 indicating 1-3 leaves rolled and yellowed, 2 indicating more than 3 leaves rolled and deformed, 3 showing chlorosis and early plant wilting, 4 indicating necrosis and entire plant wilting, and 5 representing dead or near-death plants (Table 2). For tomato Fusarium wilt, the scale ranged from 0 to 4 as described by Kwak *et al*., 2018: 0 representing healthy plants, 1 indicating 1-25% leaves wilted, 2 indicating 26-50% leaves wilted, 3 indicating 51-75% leaves wilted, and 4 indicating 75-100% leaves wilted. The percentage of disease severity index was calculated using the below formula.

$$\text{Percent disease severity index}=\frac{\text{Sum of indivisual rating}}{\text{No. of infected plant observed}\times \text{Maximum disease scale}}\times 100$$

The strawberries and tomatoes phenotypes and disease severity index measured for each treatment group were analysed for significance using Kruskal-Wallis Rank Sum Test [45], and significant differences were confirmed with a *p*-value of less than 0.05. Post-hoc comparisons using the Conover Test were then conducted to display differences in disease suppression efficacy among treatment groups [46].

## Results

### *C. fusca* CHK0059 Triggered Microbial Community Structure Change in Strawberry Microbiota Fraction

Prior to the observation of changes in microbiota community over time in MF treated with each *Chlorella* substance, the extracted strawberry rhizosphere MF stock was treated, and then the struct of community changed over time *in vitro* was verified. As a result of checking the microbial density concentration of each treatment group by time, no significant difference was found in the microbial concentration between each treatment groups up to 12 h (Fig. S1). However, when differences began to appear at 24 h after 12 h, that were a significant difference between each treatment group at 36 h. Especially, D-mannitol 2% treatment maintained more than *Chlorella* 10^7^ treatment until 36 h after treatment, while in the sterilized *Chlorella* and living *Chlorella* treatments (Chlorella 10^6^ treatment, *Chlorella* 10^7^ treatment) at different concentrations, the cell density of microorganisms found in the medium decreased over time to about *Chlorella* 10^6^ treatment. We assumed that each treated substance would have affected the density of microorganisms present in the strawberry MF. At 12 h and 36 h timepoints where significant differences occurred were selected and the distribution diversity of microorganisms present.

### Rhizosphere Microbial Community Diversity by CHK0059 Substances

To determine how the microbial community responses when CHK0059, methanol extract, and D-mannitol were applied in strawberry rhizosphere, microbiome analysis was performed by treating the substances in a strawberry rhizosphere MF. Alpha-diversity was analysed to confirm the diversities within microbiota communities between each treatment (Fig. S2). Based on ASV, in observed, methanol extract 0.01x and D-mannitol 0.2% treatment showed significantly diversity in the community between 12 h and 36 hr based on *p*-value < 0.05. However, according to the Shannon and Simpson index, all treatments exhibited significantly diversity in community between 12 h and 36 h. The significant difference in diversity observed in all treatments based on Shannon and Simpson index suggested that, over time, the microbial communities underwent a shift towards a more balanced distribution of species, even if the total number of species did not change significantly. For the Methanol extract 0.01x and D-mannitol 0.2% treatments, the observed significant differences may indicate that these treatments specifically influenced both the presence of new species and their relative abundances within the community. Through this, the microbiota diversities were enhanced by CHK0059 increased, due to the substance treatments compared to the untreated group. But in the case of other treatments, the diversity of microbiota within the community changed similarly to the untreated group (Fig. S2).

Beta-diversity was an analysis representing diversity among microbial communities (Fig. S3). In the beta diversity, the differences in distribution between microbiota community in each treatment were confirmed. At the family level top 10 ASVs was distributed at the highest abundance in all treatments. The top 10 microorganisms in all treatments were Pseudmonadaceae, Sphingobacteriaceae, Micrococcaceae, Chitinophagaceae, Kaistiaceae, Commonadaceae, Sphingomonadaceae, Rhizobiaceae, Rhodanobacteraceae, Alcaligenaceae. Table S1 represented which microorganisms are most distributed in each treatment by ASV numbers.

**Fig. 2:**
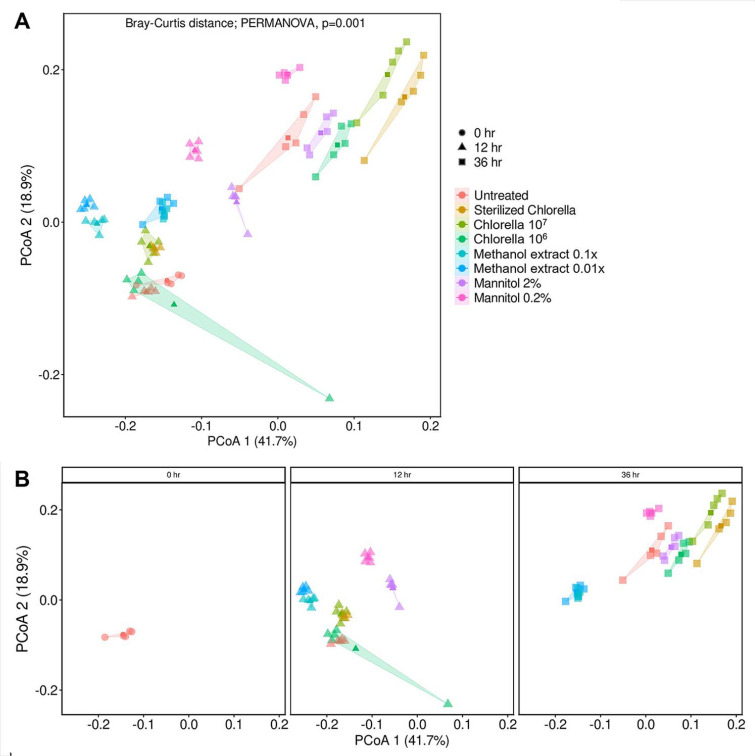
Principal coordinate analysis (PCoA) of each treatment. Dissimilarity comparison of the microbial community was based on Bray-Curtis dissimilarity metrics, revealing significant differences between each treatment. (**A**) Diversity distribution among treatments of all samples. (**B**) Diversity distribution of each treatment over time (12 h, 36 h). To analyze a wide range of distribution distances, compare by adding time 0 as a control. Statistical analysis was conducted by permutational analysis of variance (PERMANOVA).

PCoA analysis was conducted to observe how the distribution of microorganisms presents in each treatment changes over time by converting the distribution between microbial communities identified by beta-diversity into distance (Fig. 2A). Based on the PCoA analysis, the microbiota communities in the untreated control, sterilized *Chlorella*, and living *Chlorella* treatment groups exhibited similar distributions at 12 h (Fig. 2B). In the *Chlorella* 10^6^ treatment, a sample showed a different microbial community, which may appear to be a technical error. Methanol extract treatments, including methanol extract 0.1x and methanol extract 0.01x, showed similar microbial distributions at 12 h. Interestingly, in the D-mannitol treatment groups, including D-mannitol 2% and D-mannitol 0.2%, there was some distance observed in microbial distribution compared to other groups treated with the same substance at 12 h. However, overall, treatments treated with D-mannitol (D-mannitol 2% and D-mannitol 0.2%) showed similar diversity in microbial distributions over time. The results suggested that the treatments with the same substance at different concentrations exhibited similar microbial community structure. Notably, the untreated control group microbial communities showed those treated with CHK0059, while treatments with methanol extract and D-mannitol resulted in distinct microbial community compositions based on the respective substances treated (Fig. 2B).

At 36 h, significant changes in microbial community distribution were observed compared to 12 h, except for the methanol extract treatment groups. Particularly, in the D-mannitol treatment group, showed different the communities compared to CHK0059 at 12 h, D-mannitol 2% treatment exhibited microbial community changes like those of CHK0059 at 36 h. This indicated that over time, the microbial community composition in the D-mannitol 2% treatment becomes similar to CHK0059 treatment (Fig. 2).

### Keystone Taxa Response to CHK0059 in Rhizosphere MF

Additionally, the response of keystone taxa, including *Pseudomonas*, *Duganella*, *Rhizomicrobium*, and *Brevibacterium*, to the substances such as *Chlorella*, methanol extract, and D-mannitol were examined. In each sample, the keystone taxa identical to the ASV sequences identified in the previous study [36, 37], and their relative abundances were determined. Upon confirming the relative abundance of the keystone taxa, *Pseudomonas* was the only keystone taxon present among the four strains identified in tube. The other three species were not detected in the extracted strawberry rhizosphere MF.

Consequently, the relative abundance of *Pseudomonas* was compared across different treatment groups over time to assess its reaction to *Chlorella*, methanol extract, and D-mannitol. The results showed that the methanol extract treatments (0.1× and 0.01×) exhibited the highest relative abundance of *Pseudomonas* at 12 h (Fig. S4A), followed by D-mannitol 0.2%, D-mannitol 2%, and the untreated. Conversely, treatments with different concentrations of CHK0059 (sterilized *Chlorella*, *Chlorella* 10^7^, and *Chlorella* 10^6^) showed lower *Pseudomonas* abundance. At 36 h, D-mannitol 2% showed the highest *Pseudomonas* abundance (Fig. S4B), followed by methanol extract 0.1× and 0.01×. Respectively, treatments directly involving *Chlorella* consistently showed lower *Pseudomonas* abundance compared to other treatments. Over time, a decreasing trend in *Pseudomonas* abundance was observed in the sterilized *Chlorella*, *Chlorella* 10^7^, and *Chlorella* 10^6^ treatments. In contrast, an increasing trend of the *Pseudomonas* in abundance was observed in the D-mannitol 2% treatment from 12 to 36 h. These findings suggested that the keystone taxon *Pseudomonas* reacts to *Chlorella* and D-mannitol 2% treatments.

### PGP Activities of Keystone Taxa

To determine the effect on plants when treated with microorganisms capable of various biological roles, even at low relative abundance. Before treating the plants, we first confirmed the plant growth-promoting (PGP) activity of SynCom strains in the lab (Table S2 and Fig. S5). The PGP activity of the four SynCom strains (8C3D12, FT92W, 6C7F4, REN4) was assessed using a PGP activity assay. The results indicated that cellulase and chitinase activities were not detected in any of the four strains. The 8C3D12 strain exhibited only protease activity, while the REN4 strain demonstrated siderophore production and indole-3-acetic acid (IAA) production abilities. The FT92W and the 6C7F4 strains showed multiple PGP activities. Specifically, the strains had nitrogen fixation ability, and siderophore production ability. Additionally, the 6C7F4 strain had protease activity and was the only strain among the four strains to exhibit phosphate solubilization ability. Given the distinct PGP activities of the four strains, we hypothesized that a mixed treatment of these strains with plants would result in the expression of various PGP abilities.

### *In vivo* PGP Activities of Keystone Taxa in Strawberry and Tomato

After treating the plants with keystone taxa in SynCom 10^6^ CFU/ml, *F. oxysporum* f. sp. *fragariae* F9 (F9) 10^5^ CFU/ml was applied to determine its ability to suppress plant diseases. Before comparing the disease suppression abilities, the growth parameters of each treatment were evaluated to assess the effect of the SynCom strains on the plants. Comparing the number of leaves, shoot length, shoot weight, root length, root weight, and chlorophyll content are presented (Fig. S6). The PGP measurement values for each treatment group are summarized in Tables S3 and S4.

First, the PGP activity of SynCom strains in strawberries was evaluated. No significant difference was observed in the number of leaves between the untreated and other treatments (Fig. S6). However, all treatments, except the untreated treatment, showed an increase in shoot length, with the highest shoot length observed in the Treat3 group treated with 6C7F4, averaging 18.47 cm. Treatments with FT92W (Treat2) and SynCom (Treat8) also had long shoot lengths. Compared to the F treatment group, treatments with single and mixed SynCom (Treat1 to Treat9) exhibited increased shoot lengths. Treat10, treated only with 2% D-mannitol, had a shoot length like the F treatment (Fig. S6).

In terms of shoot weight, the untreated group and single strain treatments (Treat1 to Treat4) had shoot weight of 1.85 g, 1.53 g, 1.38 g, 2.24 g, 1 g respectively, compared to the *Fusarium* treatment group (0.43 g). Treat3 had the heaviest shoot weight, averaging 2.24 g. The only SynCom treatment (Treat8) had an average shoot weight of 1.47 g, more than 3.4 times that of the Fusarium treatment group. *Fusarium* and Treat10 treatments had lower shoot weights (Fig. S6). For root length, Treat2 had the longest root length at 20 cm, followed by Treat4 (19 cm), Treat3 (18.67 cm), Treat8 (16 cm), and Treat5 (15 cm). The untreated group (12.33 cm), Fusarium treatment, and Treat6 (13 cm) treated with a mixture of 8C3D12 and FT92W strains had longer root lengths than the Fusarium treatment (10.77 cm) but were relatively short among the treatments (Fig. S6). Root weight was heaviest in the untreated group (4.56 g), followed by Treat2 (1.13 g), Treat3 (0.83 g), Treat7 (0.87 g), and Treat8 (0.76 g) (Fig. S5E). Chlorophyll content was highest in the Fusarium treatment (18.29 μg/cm^2^), followed by Treat1 (16.89 μg/cm^2^). Treat8 and Treat9, treated with all SynCom strains, also had high chlorophyll content, averaging 16.92 μg/cm^2^ and 16.08 μg/cm^2^, respectively. Treat5, treated with CHK0059, which aids photosynthesis, had lower chlorophyll content than the F treatment but higher than the untreated treatment (15.76 μg/cm^2^ compared to 14.04 μg/cm^2^) (Fig. S6).

PGP activity in tomatoes was also observed to determine whether SynCom strains could be applied to hosts other than strawberries. Treat8 had the highest number of leaves, followed by Treat6, Treat9, Treat10, and the untreated group, but Treat3 showed the fewest leaves (Tables S3 and Fig. S7). The untreated group had the longest shoot length (42.5 cm), while the Fusarium treatment group had the shortest (27.83 cm). Among the SynCom-treated groups, Treat9 had the second longest shoot length (38.67 cm), followed by Treat6 (36.33 cm).

Shoot weight was highest in the untreated group (16.07 g). The F treatment had the lowest shoot weight (4.9 g) (Fig. S7). Treat6 had the highest shoot weight among the strain treatments (average 13.4 g). Treat2 and Treat9 were lower than the untreated group but were 56.1% and 57.4% heavier than the F treatment. Root length was longest in the untreated group (20 cm), with the F treatment having the shortest length (Fig. S7). The untreated group and Treat6 treated groups had longer roots length (17.67 cm). Treat1 (11.67 cm) and Treat2 (12 cm) had root lengths 52.87% and 52.17% longer than the F treatment. Treat9 (15.33 cm), treated with SynCom strain and CHK0059, had the next longest root length. Root weight was highest in Treat6 (0.57 g) (Fig. S7). F (0.06 g) and Treat10 (0.07 g) had the lowest root weights. Treat5 (0.28 g), Treat7 (0.36 g), and Treat9 (0.39 g), treated with CHK0059, had relatively heavy root weights. Chlorophyll content was highest in F (8.97 μg/cm^2^) and Treat1 (8.08 μg/cm^2^). Treat3 (3.34 μg/cm^2^) had the lowest chlorophyll content, followed by Treat2 (5.96 μg/cm^2^), Treat4 (7.98 μg/cm^2^), Treat8 (7.74 μg/cm^2^), and Treat10 (5.88 μg/cm^2^), which was similar to the untreated group (6.48 μg/cm^2^) (Fig. S7). These results confirm that despite F treatment, the SynCom strain treatments (single and mixed) showed higher PGP activity than the F treatment group. Notably, treatments with FT92W or 6C7F4 strains alone exhibited higher growth rates and demonstrated that SynCom interacting with CHK0059 can improve plant growth, having a positive effect when it applied to plants.

### Biological Activities of the SynCom in Strawberry and Tomato *in vivo*

**Fig. 3:**
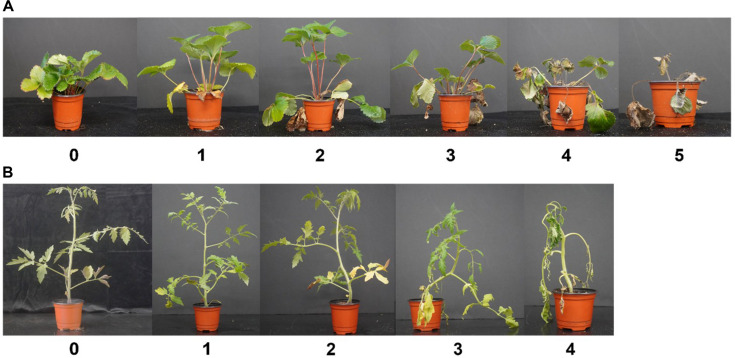
Disease severity scale of *F. oxysporum*. (**A**) *F. oxysporum* f. sp. *fragariae* on strawberry and (**B**) *F. oxysporum* f. sp. *lycopersici* on tomato. Disease index was measured each week after the pathogen treated and severity of wilt were evaluated. (**A**) The severity of strawberry Fusarium wilt disease includes the necrosis of the roots. Strawberry disease severity scales were represented by 0: Healthy, 1: 1~3 leaves rolled and yellowed, 2: more than 3 leaves rolled and deformed, 3: Chlorosis and early plant wilting, 4: Necrosis and entire plant wilting, 5: Dead or nearly so. (**B**) Tomato disease severity scales were represented by 0: Healthy, 1: 1~25% leaves wilted, 2: 26~50% leaves wilted, 3: 51~75% leaves wilted, 4: 75~100% leaves wilted. The disease index measured all the plants in each treatment (*n* = 10).

**Fig. 4:**
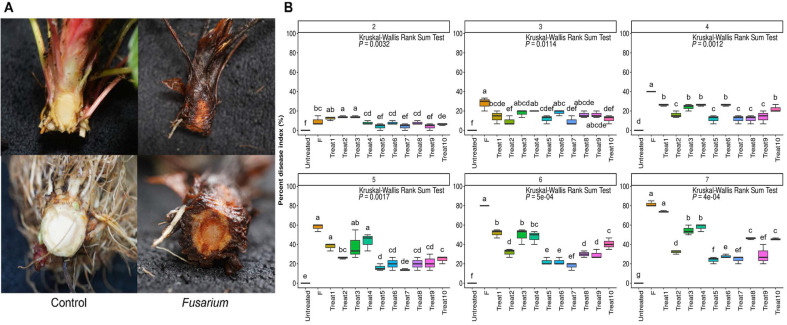
Disease index percentages of Strawberry by *F. oxysporum* f. sp. *fragariae*. Disease index was measured each week and severity of wilt were evaluated with the scales (scales: 0~5). Symptoms of Fusarium wilt disease were observed until the 7 weeks. (**A**) Strawberry crowns cut, exposing vascular discoloration in *Fusarium* treatment. (**B**) The percentage of DI (%) of strawberry Fusarium wilt disease includes the necrosis of the roots. Each 10 plants were divided into 3 groups and each index was measured (*n* = 10). F: *F. oxysporum* f. sp. *fragariae* F9 (F9), Treat1: 8C3D12 + F9; Treat2: FT92W + F9, Treat3: 6C7F4 + F9, Treat4: REN4+ F9, Treat5: CHK0059 + F9, Treat6: 8C3D12 + FT92W + F9, Treat7: 8C3D12 + FT92W + CHK0059 + F9, Treat8: SynCom + F9, Treat9: SynCom + CHK0059 + F9, Treat10: D-mannitol 2% + F9. The numbers above each graph represent weekly intervals. Disease severity was indicated with different alphabets as significant difference according Kruskal-Wallis Rank Sum Test followed by Conover test using ‘bh’ method (*p* < 0.05). The box plots were calculated with the ggplot2 package of the R (4.3.2).

To compare the antifungal activity of SynCom strains in plants, strawberries and tomatoes were treated with SynCom and *F. oxysporum* to assess the antifungal efficacy of SynCom members. For strawberry, *F. oxysporum* f. sp. *fragariae* was inoculated (10^5^ CFU/ml), and the disease index (DI) was measured weekly until the 7^th^ week to determine the percentage of disease severity (Fig. 3). To verify disease occurrence, the conduits of the untreated group and the F treatment group were cut to check for signs of disease (Fig. 4A). In the F treatment group, leaf desiccation and discoloration of the ducts were observed, indicating the occurrence of Fusarium wilt disease caused by *F. oxysporum* f. sp. *fragariae* F9. Therefore, the DI percentage was measured weekly following the treatment with the F9. During the first two weeks, the F treatment showed a low disease index. However, by the 3^rd^ week, symptoms began to appear in the F treatment, and the disease progression increased weekly (Fig. 4B). By the 5^th^ week, the disease index percentage in the F treatment group rose up to approximately 60%, while the SynCom treatment group showed a disease index 20% percentage, demonstrating a significant difference in disease incidence between the F and SynCom treatments. After 7 weeks, the F and Treat1 groups exhibited the highest co-occurrence rate of over 80%, whereas Treat5, Treat6, Treat7, and Treat9 showed co-occurrence rates of 50-60%, which were lower than the F group. Overall, compared to the group treated with the SynCom strain alone, Treat5 (CHK0059), Treat6 (8C3D12 and FT92W), Treat7 (8C3D12 and FT92W), and Treat9 (SynCom strain and CHK0059) had low disease index percentage of 23.9%, 27.3%, 24%, and 28.6%, respectively. These results suggest that CHK0059 treatment can inhibit *Fusarium oxysporum* f. sp. *fragariae* F9.

**Fig. 5:**
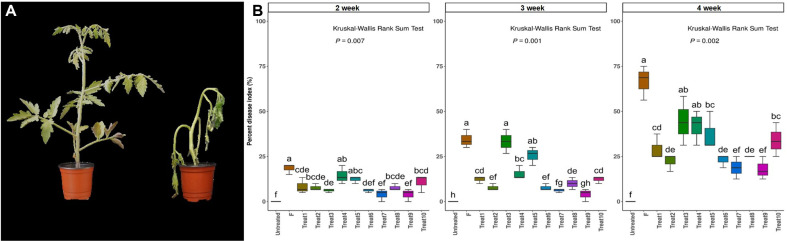
Percentage of disease index in tomato infected by *F. oxysporum* f. sp. *lycopersici*. Disease index was measured each week and severity of wilt were evaluated with the scales (scales: 0~4). (**A**) Symptom of infected tomato plant (**B**) The percentage of DI (%) of tomato Fusarium wilt disease. Symptoms of Fusarium wilt disease were observed until the 4 weeks. Disease severity values were measured by grouping 10 plants in each treatment into 3 groups. The numbers above each graph represent each week. F: *F. oxysporum* f. sp. *lycopersici*, Treat1: 8C3D12 + *F. oxysporum* f. sp. *lycopersici*; Treat2: FT92W + *F. oxysporum* f. sp. *lycopersici*, Treat3: 6C7F4 + *F. oxysporum* f. sp. *lycopersici*, Treat4: REN4+ *F. oxysporum* f. sp. *lycopersici*, Treat5: CHK0059 + *F. oxysporum* f. sp. *lycopersici*, Treat6: 8C3D12 + FT92W + *F. oxysporum* f. sp. *lycopersici*, Treat7: 8C3D12 + FT92W + CHK0059 + *F. oxysporum* f. sp. *lycopersici*, Treat8: SynCom + *F. oxysporum* f. sp. *lycopersici*, Treat9: SynCom + CHK0059 + *F. oxysporum* f. sp. *lycopersici*, Treat10: D-mannitol 2% + *F. oxysporum* f. sp. *lycopersici*. Disease severity was indicated with different alphabets as significant difference according Kruskal-Wallis Rank Sum Test followed by Conover test using ‘bh’ method (*p* < 0.05). The results were calculated with the ggplot2 package of the R (4.3.2).

For tomatoes, *F. oxysporum* f. sp. *lycopersici* (10^5^ CFU/ml) was applied, and disease severity was measured weekly until the 4^th^ week (Fig. 5). Symptoms began to appear in the F treatment group from the 2^nd^ week, with a noticeable difference in disease index percentages compared to other treatments. By the 3^rd^ week, F and Treat3 had higher disease index values than other treatments, and some disease outbreaks were observed in Treat5. By the 4^th^ week, the F treatment group showed the highest disease index percentage (68~75%). Treatments with single strains such as Treat3, Treat4, Treat5, Treat10, and those with CHK0059 alone or D-mannitol 2% had higher disease index percentages on average (44.4%, 41.6%, 37.5%, and 34%) next to F treatment. Conversely, Treat1, Treat2, Treat6, Treat7, Treat8, and Treat9 had lower disease index percentages, averaging 29.2%, 22.2%, 22.9%, 18.8%, 25%, and 18%, respectively. Notably, Treat7 and Treat9, which combined CHK0059 with the SynCom strain, exhibited the lowest disease index (Fig. 5B).

## Discussion

Microalgae, known as plankton, can be utilized by microorganisms [52]. Some researchers suggested that living plankton does not provide sufficient nutrients, and as algae die, they transform into organic matter that is readily assimilable, causing bacteria to concentrate in areas where algae are present [53]. *C. fusca* CHK0059 has been demonstrated to promote plant growth and suppress various plant diseases [8, 33, 34]. However, the underlying mechanisms of CHK0059 remain unclear. We hypothesized that the positive effects of *Chlorella* on plants are due to its interaction with microbiota, including keystone taxa, that associate with the plants. Our experiment aimed to investigate how the microbial community, particularly keystone taxa, responds to substances derived from *Chlorella* over time.

Studies have shown that Azotobacter and certain yeasts can thrive in the presence of living cells of *Chlorella* and *Scenedesmus* [54]. This may result from a symbiotic relationship or selective utilization of phytoplankton secretions by bacteria [55]. However, bacterial growth was more favourable in dead *C. vulgaris* and *S. obliquus*, likely due to the abundance of nutrients from autoclaved algae [54]. In our study, both autoclave-killed and living *Chlorella* treatments resulted in similar microbial concentrations and diversity. Microbial concentrations remained stable for up to 12 h but began to change between 24 h and 36 h, indicating a reaction to the treated substances. This suggests that *Chlorella*, methanol extract, and D-mannitol affected microbial diversity differently over time.

The "Observed" metric for alpha diversity specifically compares species diversity based on the number of amplicon sequence variants (ASVs). In contrast, the Shannon and Simpson indices are metrics that represent community diversity by reflecting both the richness (number of taxa) and the evenness (relative abundances of taxa) of microbial species within a sample. The Shannon index indicates species richness and evenness, while the Simpson index represents relative abundance [55]. Alpha-diversity metrics (Shannon and Simpson indices) showed significant differences across all treatments, except for methanol extract 0.01x and D-mannitol 0.2% treatment. These indices reflect both species richness and evenness, indicating changes in microbial species diversity. Community richness is a measure that estimates the total number of species present in a sample. The alpha-diversity results showed that all treatments had significant differences over time (*p* < 0.05) in the Shannon and Simpson indices, confirming changes in microbial species diversity across all treatments. Beta-diversity refers to the variability in community composition (i.e., the types of taxa observed) among samples within a given habitat [56]. Principal Coordinates Analysis (PCoA) represents the distance between all pairs of species based on their traits. Beta-diversity analysis using PCoA revealed that 2% D-mannitol treatment had shifted to a microbial distribution structure similar to that of the *Chlorella* treatments. These results can suggest that CHK0059 and D-mannitol had similar effects on microbial community composition, while methanol extract had a distinct effect.

C-NMR and ^13^C NMR spectra analysis of the CHK0059 methanol extract confirmed the presence of a long fatty acid chain and multiple carbon peaks, suggesting a high carbon content. The ^13^C NMR spectrum, particularly at 20.56-34.24 ppm, indicated 22 carbon peaks corresponding to a chain-shaped group typically found in fatty acids. Additionally, the ^13^C NMR DEPT spectrum analysis detected 2 primary carbons, 25 secondary carbons, 14 tertiary carbons, and 2 quaternary carbons, suggesting a high carbon content in the methanol extract [57]. D-mannitol, a well-known carbon source like acetate, ethanol, fructose, and lactose, has been shown to increase bacterial density by acting as a carbon source [58]. D-mannitol is decomposed by *Pseudomonas* [59]. In our experiment, *Pseudomonas* density decreased in the *Chlorella* (Sterilized *Chlorella*, *Chlorella* 10^7^, *Chlorella* 10^6^) and 0.2% D-mannitol treatments, but increased in the methanol extract (methanol extract 0.1x, methanol extract 0.01x) and 2% D-mannitol treatments, assuming to be a rapid response of acting as carbon sources. The concentration of D-mannitol can affect the cell density of *Pseudomonas putida* S12 [60]. D-mannitol accumulates within cells in response to osmotic stress. D-mannitol accumulation is associated with cell growth, and certain concentrations of mannitol can affect the physiological state of cells. For example, accumulation of mannitol can help cells better adapt to changes in osmotic pressure, which can have a positive effect on cell density. In the 2% D-mannitol treatment, 2% D-mannitol concentration can provide more energy and resources for the cells, allowing them to utilize more nutrients effectively, which can lead to increased growth and density. However, in the 0.2% D-mannitol treatment, the lower accumulation of D-mannitol may limit the positive effects on *Pseudomonas* cell growth. Furthermore, treating strawberries and tomatoes with 2% D-mannitol resulted in improved disease suppression, as evidenced by a lower disease index compared to the untreated. We attributed this result to the high expression of 'Hexitol fermentation to lactate, formate, ethanol, and acetate' observed in a previous study when CHK0059 was applied to the strawberry rhizosphere, where keystone taxa were presented [37].

Keystone taxa play crucial roles in mediating soil microbial community composition and function. Despite a decrease in *Pseudomonas* density with *Chlorella* treatment, suggesting that CHK0059 may not significantly increase the density of keystone taxa. However, while keystone taxa are typically abundant, their abundance alone does not necessarily reflect their importance to the community [24, 61]. Keystone taxa are defined by their roles in biogeochemical processes such as nitrification, denitrification, and methanogenesis [62]. For instance, *Desulfosporosinus* sp. plays a crucial role in sulfate reduction and carbon flow in peatland soils despite representing only 0.06% of the total microbial community [63]. Although these microorganisms initially showed a decreased density with *Chlorella* treatment, their biochemical characteristics suggest a close relationship with *Chlorella* due to their interactions with plants.

We hypothesized that applying keystone taxa as a SynCom would yield synergistic effects on plant growth due to interactions among the taxa. Applying keystone taxa as a SynCom to strawberries and tomatoes resulted in improved plant growth and disease suppression. Specifically, strawberries treated with the SynCom exhibited increased shoot length, root length, and total chlorophyll, while tomatoes showed increased leaf number and root weight compared to the untreated plants. The findings suggested that SynCom strains have PGP activity in various crops and that the mixed treatment may enhance this effect, similar to previous findings with synthetic communities in *Zea mays* [64]. Among the SynCom strains, *Pseudomonas* has been shown to produce volatile and non-volatile metabolites that inhibit pathogens [65, 66]. *Pseudoduganella*, which is genetically similar to genera such as *Duganella* and *Massilia*, can produce violacein, solubilize phosphorus, potassium, and zinc, and exhibit PGP activity [67]. *Brevibacterium*, another SynCom strain, has been identified as a plant growth-promoting rhizobacterium (PGPR) candidate due to indole 3-acetic acid production, phosphate solubilization, and biofilm formation abilities, which enhance root length and seedling weight [68]. These activities suggest that keystone taxa positively effect on plant growth. When SynCom was applied to strawberries to evaluate its effect on *Fusarium* pathogens, treatments Treat2, Treat5, Treat6, Treat7, and Treat9 showed reduced disease incidence. Similarly, in tomatoes, Treat1, Treat2, Treat6, Treat7, Treat8, and Treat9 also exhibited lower disease incidence. Among these, FT92W demonstrated high antifungal activity against *F. oxysporum* in strawberries, especially when combined with other SynCom strains. Furthermore, the combination of SynCom strains with CHK0059 (Treat9) resulted in higher disease inhibitory activity compared to SynCom alone, indicating enhanced pathogen suppression. These results align with previous studies showing that SynCom treatments often outperform single-strain treatments in antifungal activity [42]

According to these experiments, we confirmed that SynCom strains exhibited PGP activity and antifungal properties, enhancing pathogen suppression when combined with CHK0059. These findings align with previous studies showing that SynCom treatments often outperform single-strain treatments in antifungal activity [42]. Our experiments confirmed that 2% D-mannitol produces effects similar to CHK0059 on microorganisms. Keystone taxa interacting with CHK0059 exhibited PGP activities, and when applied as SynCom, they induced synergistic effects on plant growth and disease suppression. Further experiments are needed to genetically confirm how keystone taxa respond to CHK0059 and D-mannitol in plants. Our results support the hypothesis that microbiota play a significant role in the mechanism of action for *Chlorella*, as key microorganisms demonstrated effects on plants similar to *Chlorella*.

## Conclusion

To determine the substances of *C. fusca* CHK0059 that influence the plant's microbial community and keystone taxa, we treated various CHK0059 substances and observed the microbial community response over time. Notably, the microbial community structure became similar when CHK0059 and 2% D-mannitol were treated. This suggests that the hexitol fermentation metabolic pathway of the core microorganisms, previously identified, is likely upregulated by CHK0059 treatment. Further investigation of the biological characteristics of CHK0059 keystone taxa revealed that they promote plant growth and suppress Fusarium wilt disease in both strawberries and tomatoes. Additionally, treating plants with these keystone taxa in the SynCom demonstrated a synergistic effect, resulting in more substantial plant growth promotion and disease suppression than treatments with individual core microorganisms. This highlights the potential of CHK0059 and its associated keystone taxa as effective agents for enhancing plant health and resilience

## Supplemental Materials

Supplementary data for this paper are available on-line only at http://jmb.or.kr.



## Figures and Tables

**Table 1:** Information of CHK0059 keystone taxa used in this study.

Origin	ASV	Identification	NCBI Blast	Distribution of strains	Reference
Strawberry Rhizosphere	seq_1	Pseudomonas	*Pseudomonas* sp. (Accession No. PQ555449)	*Pseudomonas* sp. 8C3D12 (isolate)	Lee *et al*., 2025
Strawberry Rhizosphere	seq_86	Duganella	*Pseudoduganella rivuli* FT92W (Accession No. NR_180844.1)	*Pseudoduganella rivuli* FT92W (KACC 21474)	Lee *et al*., 2025
Arabidopsis Rhizosphere	seq_111	Pseudomonas	*Pseudomonas* sp. (Accession No. PQ555450)	*Pseudomonas* sp. 6C7F4 (isolate)	Cho *et al*., 2022
Arabidopsis Rhizosphere	seq_135	Brevibacterium	*Brevibacterium renqingii* strain REN4 (Accession No. NR_181446)	*Brevibacterium renqingii* strain REN4 (KCTC 49366)	Cho *et al*., 2022

**Table 2:** Disease index (severity scale) of strawberries and tomatoes treated by *F. oxysporum*

Host	Index	Symptoms	Reference
Strawberry	0	Healthy	Cha *et al*., 2015
	1	1–3 leaves rolled and yellowed	
	2	More than 3 leaves rolled and deformed	
	3	Chlorosis and early plant wilting	
	4	Necrosis and entire plant wilting	
	5	Dead or nearly so	
Tomato	0	Healthy	Kwak *et al*., 2018
	1	1 ~ 25% leaves wilted	
	2	26 ~ 50% leaves wilted	
	3	51 ~ 75% leaves wilted	
	4	76 ~ 100% leaves wilted	
