# Supplementary material for: A Keystone-Taxa SynCom Reveals Chlorella–Microbiome–Plant Communication and Enhances Suppression of Fusarium oxysporum
Source: J Microbiol Biotechnol. 2026 Apr 3;36:e2601023. doi: 10.4014/jmb.2601.01023 (PMC13066648; doi:10.4014/jmb.2601.01023)
Supplement: Supplementary file 1 [file jmb-36-e2601023-supple.pdf]

J. Microbiol. Biotechnol. 2026. 36: e2601023

https://doi.org/10.4014/jmb.2601.01023

Supplementary Tables and Figure

A Keystone-Taxa SynCom Reveals Chlorella–Microbiome–Plant Communication and Enhances Suppression of *Fusarium oxysporum*

Hwa-Jung Lee, Jin Hwan Lee, and Youn-Sig Kwak[2*]

Table S1. Identification of the top 10 ASVs in each treatment.

Treatment	Sq	phylum	class	order	family	genus	mean_sd_rel
Untreated	Sq_1	Proteobacteria	Gammaproteobacteria	Pseudomonadales	Pseudomonadaceae	*Pseudomonas*	17.92 ± 0.88
	Sq_2	Proteobacteria	Gammaproteobacteria	Pseudomonadales	Pseudomonadaceae	*Pseudomonas*	13.43 ± 0.53
	Sq_3	Proteobacteria	Gammaproteobacteria	Pseudomonadales	Pseudomonadaceae	*Pseudomonas*	9.67 ± 1.31
	Sq_4	Actinobacteriota	Actinobacteria	Micrococcales	Micrococcaceae	NA	9.36 ± 3.01
	Sq_5	Proteobacteria	Alphaproteobacteria	Rhizobiales	Kaistiaceae	Kaistia	6.74 ± 0.56
	Sq_6	Bacteroidota	Bacteroidia	Chitinophagales	Chitinophagaceae	*Arachidicoccus*	6.62 ± 1.53
	Sq_10	Bacteroidota	Bacteroidia	Sphingobacteriales	Sphingobacteriaceae	NA	5.59 ± 0.31
	Sq_9	Proteobacteria	Gammaproteobacteria	NA	NA	NA	3.95 ± 0.23
	Sq_8	Actinobacteriota	Actinobacteria	Micrococcales	Micrococcaceae	NA	3.84 ± 0.85
	Sq_7	Bacteroidota	Bacteroidia	Sphingobacteriales	Sphingobacteriaceae	*Pedobacter*	3.41 ± 0.11
Sterilized Chlorella	Sq_2	Proteobacteria	Gammaproteobacteria	Pseudomonadales	Pseudomonadaceae	*Pseudomonas*	15.77 ± 0.55
	Sq_1	Proteobacteria	Gammaproteobacteria	Pseudomonadales	Pseudomonadaceae	*Pseudomonas*	15.04 ± 0.28
	Sq_4	Actinobacteriota	Actinobacteria	Micrococcales	Micrococcaceae	NA	11.45 ± 2.29
	Sq_3	Proteobacteria	Gammaproteobacteria	Pseudomonadales	Pseudomonadaceae	*Pseudomonas*	8.50 ± 0.36
	Sq_6	Bacteroidota	Bacteroidia	Chitinophagales	Chitinophagaceae	*Arachidicoccus*	6.72 ± 0.26
	Sq_22	Bacteroidota	Bacteroidia	Sphingobacteriales	Sphingobacteriaceae	*Mucilaginibacter*	6.49 ± 1.04

	Sq_20	Bacteroidota	Bacteroidia	Chitinophagales	Chitinophagaceae	NA	5.92 ± 0.88
	Sq_10	Bacteroidota	Bacteroidia	Sphingobacteriales	Sphingobacteriaceae	NA	4.70 ± 0.41
	Sq_8	Actinobacteriota	Actinobacteria	Micrococcales	Micrococcaceae	NA	4.64 ± 0.59
	Sq_5	Proteobacteria	Alphaproteobacteria	Rhizobiales	Kaistiaceae	*Kaistia*	4.31 ± 0.42
Chlorella 10^7	Sq_2	Proteobacteria	Gammaproteobacteria	Pseudomonadales	Pseudomonadaceae	*Pseudomonas*	16.04 ± 0.61
	Sq_24	Proteobacteria	Gammaproteobacteria	Burkholderiales	Comamonadaceae	NA	15.27 ± 1.29
	Sq_1	Proteobacteria	Gammaproteobacteria	Pseudomonadales	Pseudomonadaceae	*Pseudomonas*	14.88 ± 0.81
	Sq_4	Actinobacteriota	Actinobacteria	Micrococcales	Micrococcaceae	NA	9.88 ± 2.53
	Sq_3	Proteobacteria	Gammaproteobacteria	Pseudomonadales	Pseudomonadaceae	*Pseudomonas*	8.77 ± 0.27
	Sq_5	Proteobacteria	Alphaproteobacteria	Rhizobiales	Kaistiaceae	*Kaistia*	5.90 ± 0.39
	Sq_8	Actinobacteriota	Actinobacteria	Micrococcales	Micrococcaceae	NA	4.53 ± 0.74
	Sq_6	Bacteroidota	Bacteroidia	Chitinophagales	Chitinophagaceae	*Arachidicoccus*	4.38 ± 0.42
	Sq_10	Bacteroidota	Bacteroidia	Sphingobacteriales	Sphingobacteriaceae	NA	4.35 ± 0.33
	Sq_7	Bacteroidota	Bacteroidia	Sphingobacteriales	Sphingobacteriaceae	*Pedobacter*	4.33 ± 0.29
Chlorella 10^6	Sq_1	Proteobacteria	Gammaproteobacteria	Pseudomonadales	Pseudomonadaceae	*Pseudomonas*	18.40 ± 4.02
	Sq_2	Proteobacteria	Gammaproteobacteria	Pseudomonadales	Pseudomonadaceae	*Pseudomonas*	12.87 ± 3.41
	Sq_4	Actinobacteriota	Actinobacteria	Micrococcales	Micrococcaceae	NA	10.09 ± 2.74
	Sq_3	Proteobacteria	Gammaproteobacteria	Pseudomonadales	Pseudomonadaceae	*Pseudomonas*	8.91 ± 1.73
	Sq_5	Proteobacteria	Alphaproteobacteria	Rhizobiales	Kaistiaceae	*Kaistia*	6.60 ± 0.51
	Sq_6	Bacteroidota	Bacteroidia	Chitinophagales	Chitinophagaceae	*Arachidicoccus*	5.78 ± 2.68
	Sq_10	Bacteroidota	Bacteroidia	Sphingobacteriales	Sphingobacteriaceae	NA	5.64 ± 3.03
	Sq_8	Actinobacteriota	Actinobacteria	Micrococcales	Micrococcaceae	NA	5.26 ± 0.65
	Sq_9	Proteobacteria	Gammaproteobacteria	NA	NA	NA	2.93 ± 0.54
	Sq_7	Bacteroidota	Bacteroidia	Sphingobacteriales	Sphingobacteriaceae	*Pedobacter*	2.66 ± 1.17
Methanol extract 0.1x	Sq_2	Proteobacteria	Gammaproteobacteria	Pseudomonadales	Pseudomonadaceae	*Pseudomonas*	21.25 ± 1.04
	Sq_1	Proteobacteria	Gammaproteobacteria	Pseudomonadales	Pseudomonadaceae	*Pseudomonas*	15.50 ± 0.58
	Sq_7	Bacteroidota	Bacteroidia	Sphingobacteriales	Sphingobacteriaceae	*Pedobacter*	7.79 ± 0.24
	Sq_3	Proteobacteria	Gammaproteobacteria	Pseudomonadales	Pseudomonadaceae	*Pseudomonas*	7.10 ± 0.21
	Sq_6	Bacteroidota	Bacteroidia	Chitinophagales	Chitinophagaceae	*Arachidicoccus*	7.10 ± 0.34
	Sq_10	Bacteroidota	Bacteroidia	Sphingobacteriales	Sphingobacteriaceae	NA	6.77 ± 0.17
	Sq_9	Proteobacteria	Gammaproteobacteria	NA	NA	NA	5.23 ± 0.22
	Sq_4	Actinobacteriota	Actinobacteria	Micrococcales	Micrococcaceae	NA	4.13 ± 0.48

	Sq_11	Proteobacteria	Gammaproteobacteria	Pseudomonadales	Pseudomonadaceae	*Pseudomonas*	4.09 ± 0.14
	Sq_8	Actinobacteriota	Actinobacteria	Micrococcales	Micrococcaceae	NA	2.87 ± 0.12
Methanol extract 0.01x	Sq_2	Proteobacteria	Gammaproteobacteria	Pseudomonadales	Pseudomonadaceae	*Pseudomonas*	21.60 ± 0.74
	Sq_1	Proteobacteria	Gammaproteobacteria	Pseudomonadales	Pseudomonadaceae	*Pseudomonas*	13.41 ± 0.50
	Sq_7	Bacteroidota	Bacteroidia	Sphingobacteriales	Sphingobacteriaceae	*Pedobacter*	13.41 ± 0.50
	Sq_3	Proteobacteria	Gammaproteobacteria	Pseudomonadales	Pseudomonadaceae	*Pseudomonas*	7.59 ± 0.39
	Sq_10	Bacteroidota	Bacteroidia	Sphingobacteriales	Sphingobacteriaceae	NA	7.36 ± 0.24
	Sq_6	Bacteroidota	Bacteroidia	Chitinophagales	Chitinophagaceae	*Arachidicoccus*	7.06 ± 0.59
	Sq_9	Proteobacteria	Gammaproteobacteria	NA	NA	NA	4.99 ± 0.57
	Sq_11	Proteobacteria	Gammaproteobacteria	Pseudomonadales	Pseudomonadaceae	*Pseudomonas*	4.01 ± 0.51
	Sq_4	Actinobacteriota	Actinobacteria	Micrococcales	Micrococcaceae	NA	3.91 ± 0.42
	Sq_8	Actinobacteriota	Actinobacteria	Micrococcales	Micrococcaceae	NA	2.63 ± 0.30
Mannitol 2%	Sq_4	Actinobacteriota	Actinobacteria	Micrococcales	Micrococcaceae	NA	13.97 ± 2.59
	Sq_2	Proteobacteria	Gammaproteobacteria	Pseudomonadales	Pseudomonadaceae	*Pseudomonas*	10.18 ± 0.99
	Sq_1	Proteobacteria	Gammaproteobacteria	Pseudomonadales	Pseudomonadaceae	*Pseudomonas*	10.15 ± 1.10
	Sq_5	Proteobacteria	Alphaproteobacteria	Rhizobiales	Kaistiaceae	*Kaistia*	9.91 ± 0.42
	Sq_3	Proteobacteria	Gammaproteobacteria	Pseudomonadales	Pseudomonadaceae	*Pseudomonas*	7.55 ± 0.77
	Sq_8	Actinobacteriota	Actinobacteria	Micrococcales	Micrococcaceae	NA	7.52 ± 1.98
	Sq_6	Bacteroidota	Bacteroidia	Chitinophagales	Chitinophagaceae	*Arachidicoccus*	6.69 ± 0.66
	Sq_10	Bacteroidota	Bacteroidia	Sphingobacteriales	Sphingobacteriaceae	NA	6.57 ± 0.75
	Sq_9	Proteobacteria	Gammaproteobacteria	NA	NA	NA	5.85 ± 0.46
	Sq_11	Proteobacteria	Gammaproteobacteria	Pseudomonadales	Pseudomonadaceae	*Pseudomonas*	4.80 ± 0.37
Mannitol 0.2%	Sq_2	Proteobacteria	Gammaproteobacteria	Pseudomonadales	Pseudomonadaceae	*Pseudomonas*	21.50 ± 1.49
	Sq_4	Actinobacteriota	Actinobacteria	Micrococcales	Micrococcaceae	NA	16.60 ± 1.28
	Sq_1	Proteobacteria	Gammaproteobacteria	Pseudomonadales	Pseudomonadaceae	*Pseudomonas*	9.90 ± 0.59
	Sq_8	Actinobacteriota	Actinobacteria	Micrococcales	Micrococcaceae	NA	8.80 ± 1.13
	Sq_3	Proteobacteria	Gammaproteobacteria	Pseudomonadales	Pseudomonadaceae	*Pseudomonas*	7.70 ± 0.37
	Sq_5	Proteobacteria	Alphaproteobacteria	Rhizobiales	Kaistiaceae	*Kaistia*	6.54 ± 0.50
	Sq_9	Proteobacteria	Gammaproteobacteria	NA	NA	NA	5.08 ± 0.26
	Sq_19	Bacteroidota	Bacteroidia	Sphingobacteriales	Sphingobacteriaceae	*Mucilaginibacter*	5.06 ± 0.28
	Sq_11	Proteobacteria	Gammaproteobacteria	Pseudomonadales	Pseudomonadaceae	*Pseudomonas*	3.37 ± 0.17
	Sq_6	Bacteroidota	Bacteroidia	Chitinophagales	Chitinophagaceae	*Arachidicoccus*	3.27 ± 0.47

Table S2. *In vivo* Plant growth promoting activity of CHK0059 SynCom.

Strain	Species	Protease	Phosphate solubilization	Nitrogen fixation	Siderophore	Cellulase	Chitinase	IAA
8C3D12	*Pseudomonas* sp.	+	-	-	-	-	-	-
FT92W	*Pseudodugaenlla rivuli*	-	-	+	+	-	-	+
6C7F4	*Pseudomonas* sp.	+	+	+	+	-	-	-
REN4	*Brevibacterium renqingqii*	-	-	-	+	-	-	+

+: Positive

-: Negative

Table S3. The phenotype of the treatments after treated *F. oxysporum* f. sp. F9 to Strawberry. Values are means ± SEM (*n* = 10).

Treatment	Leaf count	Shoot length (cm)	Shoot weight (g)	Root length (cm)	Root weight (g)	Total Chlorophyll (μg/cm^2)
Untreated	10.67±1.53a	7.33 ± 2.52d	1.85 ± 0.46a	12.33 ± 0.58de	4.56 ± 1.08a	14.04 ± 0.05g
F	7.67 ± 0.58a	12.03 ± 1.00cd	0.43 ± 0.22ef	10.77 ± 2.86e	0.50 ± 0.05de	18.29 ± 0.16a
Treat1	17.00 ± 5.29a	14.27 ± 0.64bd	1.53 ± 0.55ab	15.00 ± 2.65bcd	0.55 ± 0.15de	16.89 ± 0.42ab
Treat2	10.33 ± 1.15a	15.33 ± 2.47ab	1.38 ± 0.20ab	20.00 ± 1.00a	1.13 ± 0.17ab	10.19 ± 0.1h
Treat3	13.67 ± 4.16a	18.47 ± 1.50a	2.24 ± 0.86a	18.67 ± 0.58ab	0.83 ± 0.08abc	14.43 ± 0.02f
Treat4	9.00 ± 1.00a	14.83 ± 1.53abc	1.00 ± 0.23bc	19.00 ± 1.73a	0.49 ± 0.15e	14.87 ± 0.03ef
Treat5	11.67 ± 1.53a	14.00 ± 1.00bc	0.85 ± 0.13cd	15.00 ± 1.00bcd	0.59 ± 0.12cde	15.76 ± 0.06de
Treat6	11.33 ± 3.21a	12.53 ± 2.61bcd	0.71 ± 0.29cde	13.00 ± 1.00de	0.41 ± 0.10e	14.14 ± 0.28g
Treat7	10.33 ± 0.53a	11.67 ± 2.89cd	0.61 ± 0.34def	15.17 ± 4.37cd	0.87 ± 0.19abc	13.64 ± 0.14h
Treat8	10.00 ± 1.73a	15.33 ± 0.29ab	1.47 ± 0.44ab	16.00 ± 1.00abc	0.76 ± 0.23bcd	16.92 ± 0.05bc
Treat9	11.33 ± 1.15a	13.70 ± 2.04bc	0.64 ± 0.19cdef	14.33 ± 1.15cde	0.43 ± 0.06e	16.08 ± 0.06cd
Treat10	10.00 ± 3.47a	9.43 ± 2.90d	0.38 ± 0.07f	13.83 ± 2.02cde	0.47 ± 0.24e	15.05 ± 1.09f

Alphabets indicate significant differences between the treatments. Treat1: 8C3D12 + *F. oxysporum* f. sp. F9, Treat2: FT92W + *F. oxysporum* f. sp. F9, Treat3: 6C7F4 + *F. oxysporum* f. sp. F9, Treat4: REN4 + *F. oxysporum* f. sp. F9, Treat5: CHK0059 + *F. oxysporum* f. sp. F9, Treat6: 8C3D12 + FT92W + *F. oxysporum* f. sp. F9, Treat7: 8C3D12 + FT92W + CHK0059 + *F. oxysporum* f. sp. F9, Treat8: SynCom + *F. oxysporum* f. sp. F9, Treat9: SynCom + CHK0059 + *F. oxysporum* f. sp. F9, Treat10: D-mannitol 2% + *F. oxysporum* f. sp. F9.

Table S4. The phenotypes of each treatment treated *F. oxysporum* f. sp. *lycopersici*. Values are means ± SEM (n = 10).

Treatment	Leaf count	Shoot length (cm)	Shoot weight (g)	Root length (cm)	Root weight (g)	Total Chlorophyll (µg/cm^2)
Untreated	57.33±2.31 abc	42.5±1.32 a	16.07±3.16 a	20±1 a	0.4±0.03 ab	6.48±1.26 ab
F	30.33±6.51 de	27.83±2.75 f	4.9±1.51 f	5.5±0.5 g	0.06±0.02 e	8.97±0.59 a
Treat1	53.33±3.06 abcd	31.67±0.58 ef	10.51±0.78 bcd	11.67±3.21 cde	0.26±0.05 cd	8.08±1.53 a
Treat2	47.67±10.07 cde	36±1.73 bc	11.17±2.31 abc	12±1.73 cde	0.19±0.1 de	5.96±1.36 abc
Treat3	23.67±3.79 e	29.2±1.66 f	7.27±1.07 ef	8.17±1.61 fg	0.17±0.04 de	3.34±0.9 c
Treat4	53±8.08 bcde	34±1.89 def	10.95±1.04 cde	11.67±1.44 def	0.2±0.06 cd	7.98±1.36 abc
Treat5	49.33±12.5 abcde	35±2 cd	10.9±1.01 bc	10.67±0.58 def	0.28±0.14 bcd	4.07±1.23 bc
Treat6	62±2.65 ab	36.33±1.53 abc	13.4±2.35 ab	17.67±2.08 ab	0.57±0.04 a	4.24±1.95 bc
Treat7	53±13.23 abc	35.67±5.03 cd	10.73±2.37 bc	12.67±1.53 bcd	0.36±0.06 abc	4.07±0.35 bc
Treat8	63.67±5.77 a	34.9±0.66 cd	10.48±1 cd	13.33±4.62 bcd	0.35±0.05 abc	7.74±4.36 ab
Treat9	60.67±8.50 abc	38.67±1.53 ab	11.5±0.72 abc	15.33±4.16 abc	0.39±0.21 abc	4.3±0.87 bc
Treat10	54.33±9.87 abc	33.83±1.76 cde	8.11±1.29 def	8.67±2.08 efg	0.07±0.07 e	5.88±2.61 abc

Alphabets indicate significant differences between the treatments. Treat1: 8C3D12 + *F. oxysporum* f. sp. *lycopersici*, Treat2: FT92W + *F.*

oxysporum f. sp. *lycopersici*, Treat3: 6C7F4 + *F. oxysporum* f. sp. *lycopersici*, Treat4: REN4 + *F. oxysporum* f. sp. *lycopersici*, Treat5: CHK0059 + *F. oxysporum* f. sp. *lycopersici*, Treat6: 8C3D12 + FT92W + *F. oxysporum* f. sp. *lycopersici*, Treat7: 8C3D12 + FT92W + CHK0059 + *F. oxysporum* f. sp. *lycopersici*, Treat8: SynCom+ *F. oxysporum* f. sp. *lycopersici*, Treat9: SynCom + CHK0059 + *F. oxysporum* f. sp. *lycopersici*, Treat10: D-mannitol 2% + *F. oxysporum* f. sp. *lycopersici.*

Table S5. Pairwise adonis compare test between two treatments.

Treatment	R2	F	Pr(>F)	sig	Compare test
Untreated vs Sterilized Chlorella	0.12	59.95	0.001	***	treatment
	0.75	188.11	0.001	***	timepoint
	0.09	46.86	0.001	***	treatment:timepoint
Untreated vs Chlorella 10^6	0.08	8.70	0.007	**	treatment
	0.71	37.15	0.001	***	timepoint
	0.01	1.04	0.316		treatment:timepoint
Untreated vs Chlorella 10^7	0.18	92.19	0.001	***	treatment
	0.72	187.91	0.001	***	timepoint
	0.07	35.81	0.001	***	treatment:timepoint
Untreated vs Methanol extract 0.1x	0.27	108.21	0.001	***	treatment
	0.47	92.66	0.001	***	timepoint
	0.21	80.93	0.001	***	treatment:timepoint
Untreated vs Methanol extract 0.01x	0.30	118.19	0.001	***	treatment
	0.44	87.88	0.001	***	timepoint
	0.21	81.66	0.001	***	treatment:timepoint
Untreated vs Mannitol 2%	0.29	112.18	0.001	***	treatment
	0.52	101.62	0.001	***	timepoint
	0.14	52.37	0.001	***	treatment:timepoint
Untreated vs Mannitol 0.2%	0.38	194.66	0.001	***	treatment
	0.44	112.55	0.001	***	timepoint
	0.14	74.14	0.001	***	treatment:timepoint
Sterilized Chlorella vs Chlorella 10^6	0.04	3.83	0.042	*	treatment
	0.69	74.16	0.001	***	timepoint
	0.12	12.75	0.003	**	treatment:timepoint
Sterilized Chlorella vs Chlorella 10^7	0.01	8.81	0.011	*	treatment
	0.95	696.54	0.001	***	timepoint
	0.02	11.73	0.002	**	treatment:timepoint
Sterilized Chlorella vs Methanol extract 0.1x	0.24	203.82	0.001	***	treatment
	0.41	345.84	0.001	***	timepoint
	0.33	278.37	0.001	***	treatment:timepoint
Sterilized Chlorella vs Methanol extract 0.01x	0.25	204.70	0.001	***	treatment
	0.40	320.70	0.001	***	timepoint
	0.33	266.03	0.001	***	treatment:timepoint
Sterilized Chlorella vs Mannitol 2%	0.12	68.97	0.001	***	treatment
	0.66	385.97	0.001	***	timepoint
	0.19	112.45	0.001	***	treatment:timepoint
Sterilized Chlorella vs Mannitol 0.2%	0.13	106.75	0.001	***	treatment
	0.68	548.47	0.001	***	timepoint
	0.17	135.48	0.001	***	treatment:timepoint
Chlorella 10^6 vs Chlorella 10^7	0.07	7.13	0.007	**	treatment
	0.68	68.79	0.001	***	timepoint
	0.09	9.17	0.007	**	treatment:timepoint
Chlorella 10^6 vs Methanol extract 0.1x	0.35	30.35	0.001	***	treatment
	0.31	27.43	0.001	***	timepoint
	0.16	13.79	0.001	***	treatment:timepoint
Chlorella 10^6 vs Methanol extract 0.01x	0.36	31.98	0.001	***	treatment
	0.30	26.73	0.001	***	timepoint
	0.16	14.04	0.001	***	treatment:timepoint
	0.12	8.59	0.003	**	treatment

Chlorella 10^6 vs Mannitol 2%	0.49	34.53	0.001	***	timepoint
	0.16	11.30	0.001	***	treatment:timepoint
Chlorella 10^6 vs Mannitol 0.2%	0.23	18.33	0.001	***	treatment
	0.41	33.12	0.001	***	timepoint
	0.17	13.63	0.001	***	treatment:timepoint
Chlorella 10^7 vs Methanol extract 0.1x	0.29	286.67	0.001	***	treatment
	0.40	401.26	0.001	***	timepoint
	0.29	287.88	0.001	***	treatment:timepoint
Chlorella 10^7 vs Methanol extract 0.01x	0.30	277.37	0.001	***	treatment
	0.39	364.87	0.001	***	timepoint
	0.29	270.48	0.001	***	treatment:timepoint
Chlorella 10^7 vs Mannitol 2%	0.17	105.86	0.001	***	treatment
	0.63	398.91	0.001	***	timepoint
	0.18	115.27	0.001	***	treatment:timepoint
Chlorella 10^7 vs Mannitol 0.2%	0.15	134.30	0.001	***	treatment
	0.69	631.39	0.001	***	timepoint
	0.15	138.93	0.001	***	treatment:timepoint
Methanol extract 0.1x vs Methanol extract 0.01x	0.01	3.08	0.132		treatment
	0.94	364.67	0.001	***	timepoint
	0.01	2.26	0.178		treatment:timepoint
Methanol extract 0.1x vs Mannitol 2%	0.59	447.02	0.001	***	treatment
	0.20	153.57	0.001	***	timepoint
	0.19	140.46	0.001	***	treatment:timepoint
Methanol extract 0.1x vs Mannitol 0.2%	0.57	814.36	0.001	***	treatment
	0.20	287.90	0.001	***	timepoint
	0.22	308.76	0.001	***	treatment:timepoint
Methanol extract 0.01x vs Mannitol 2%	0.60	428.17	0.001	***	treatment
	0.20	141.90	0.001	***	timepoint
	0.18	127.37	0.001	***	treatment:timepoint
Methanol extract 0.01x vs Mannitol 0.2%	0.57	688.84	0.001	***	treatment
	0.19	234.31	0.001	***	timepoint
	0.22	268.22	0.001	***	treatment:timepoint
Mannitol 2% vs Mannitol 0.2%	0.24	119.02	0.001	***	treatment
	0.64	324.78	0.001	***	timepoint
	0.09	44.60	0.001	***	treatment:timepoint

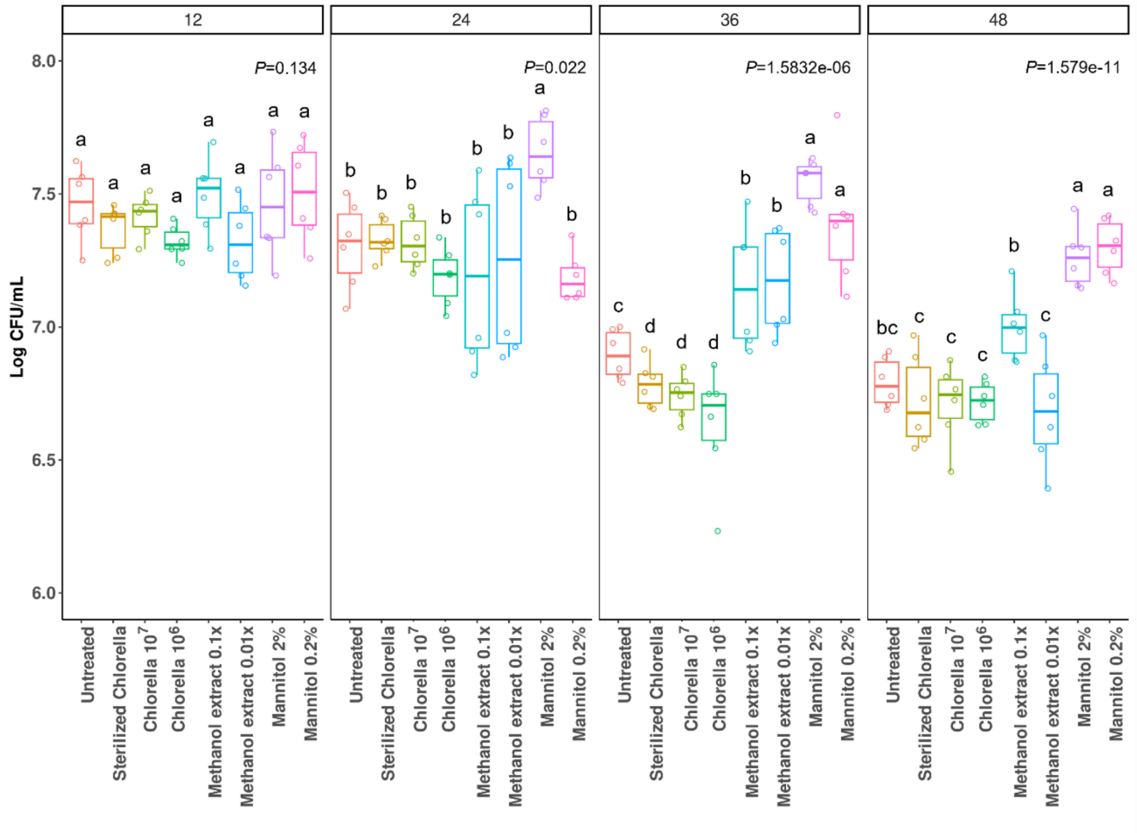

Fig. S1. Colonization of bacteria in each sample treated with different concentration of *Chlorella*, methanol extract and D-mannitol. The samples treated CHK0059 or CHK0059 extracts on the time were spread on the R2A media and incubated at 28ºC incubator for 5 days. The colony forming units (CFUs) of bacteria were counted 5 days after incubation and Statistical analysis is progressed using Tukey HSD Test and Kruskal-Wallis Rank Sum Test ($p < 0.05$). The alphabets were represented by Conover test using the 'bh' method. Error bars represent the standard deviation from three measurements (n=3). Methanol extract: CHK0059 methanol extract.

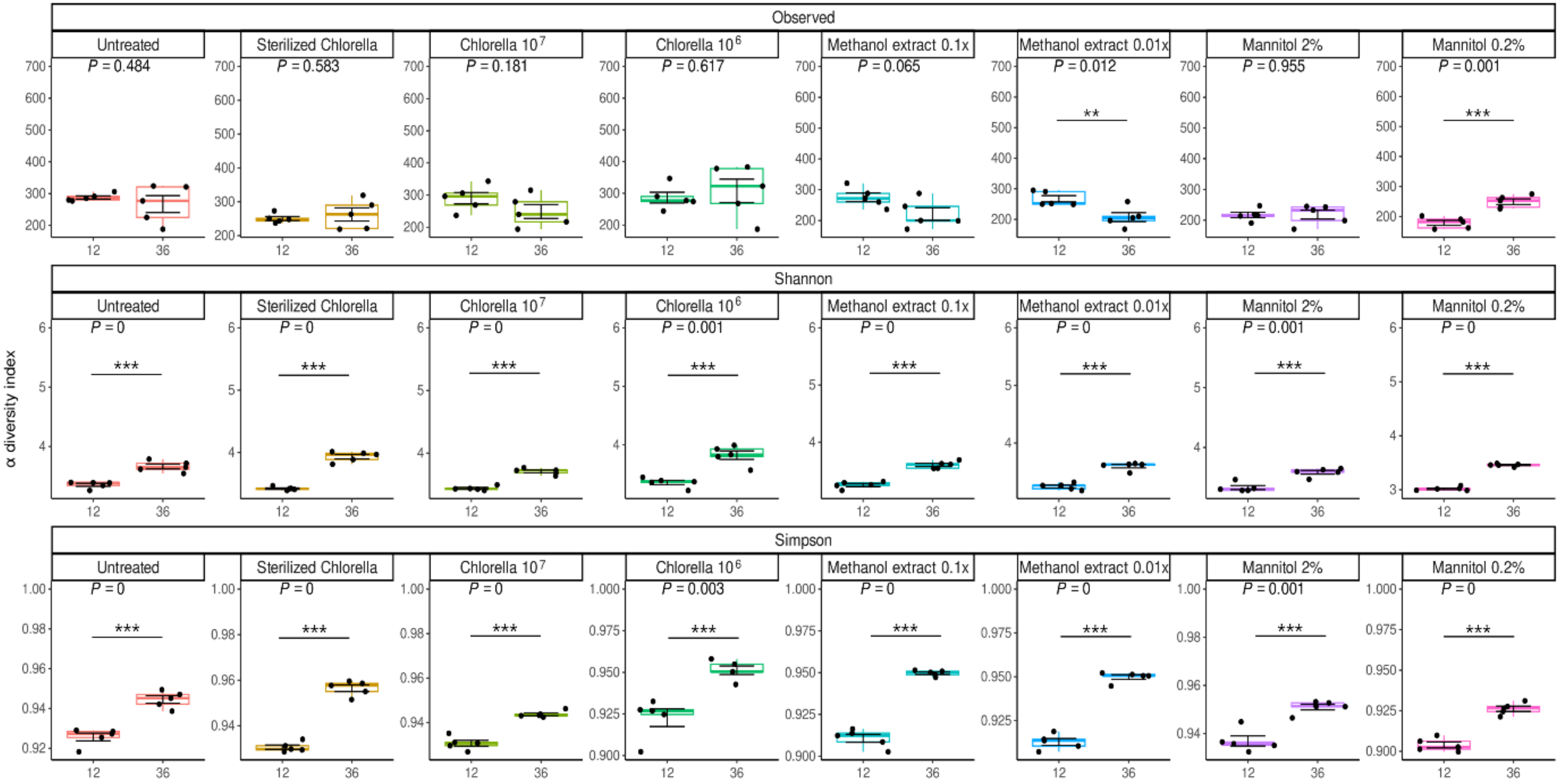

Figure S2. Alpha diversity in each sample treated with CHK0059, methanol extract and D-mannitol over time. Alpha diversity is represented by “Observed”, “Shannon index”, “Simpson index” by observed amplicon sequence variant (ASV) sequences in each

treatment. The colors represented the treatments. Significant differences in microbial diversity between 12 hr and 36 hr of each treatment were indicated with star (*). The statistical analysis was t-test between 12 hr samples and 36 hr samples in each treatment ($p < 0.05$). T-tests were calculated with ggplot2 of the R (4.3.2) package.

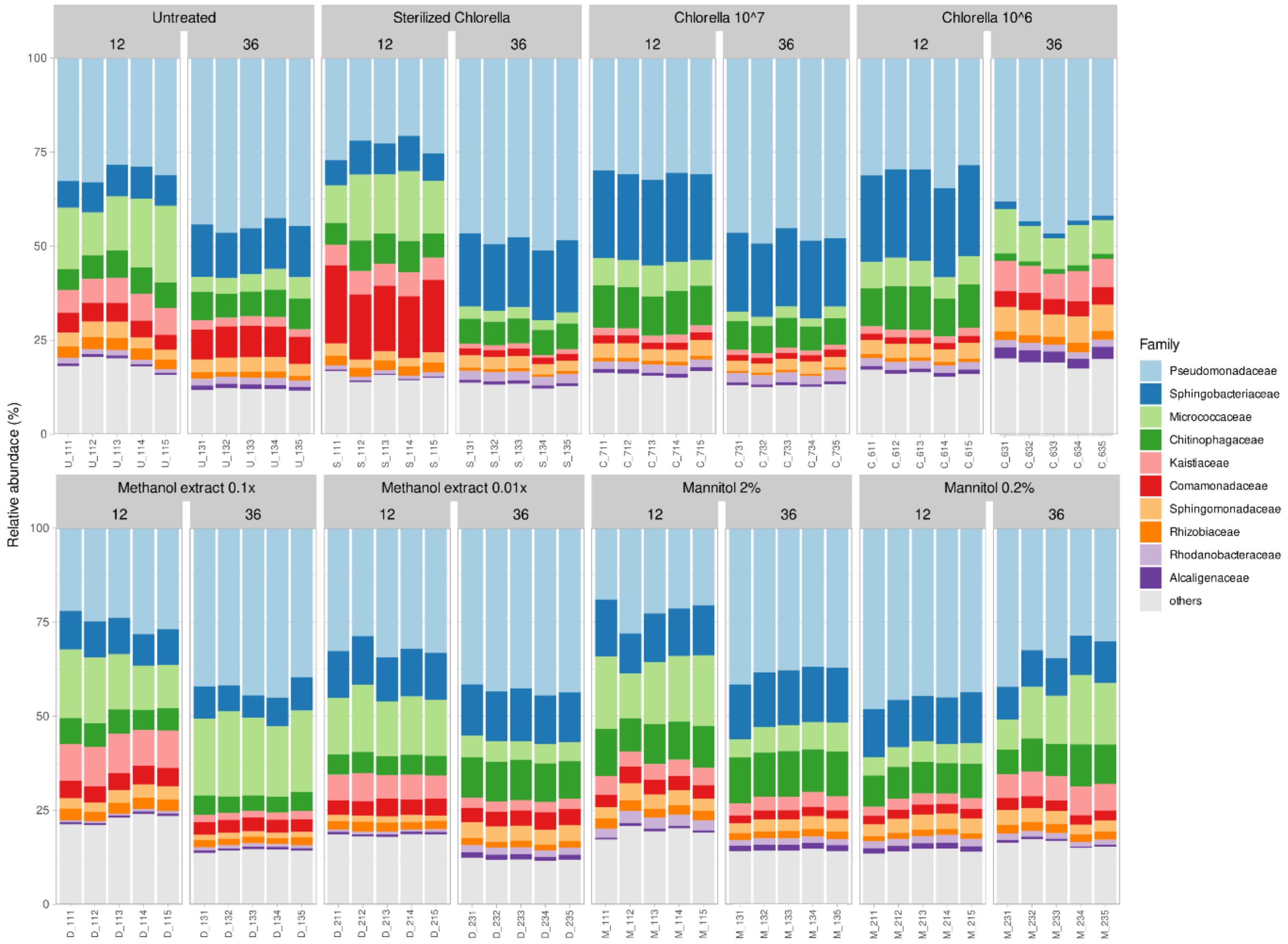

Figure S3. Relative abundance of bacteria in each treatment. The top 10 families in all samples are displayed with different colors. The x-axis indicated the name of samples. The y-axis indicated the mean relative abundance, and the color corresponds to the taxonomic group at the family levels. The identification of the top 10 families in all samples is indicated on the side.

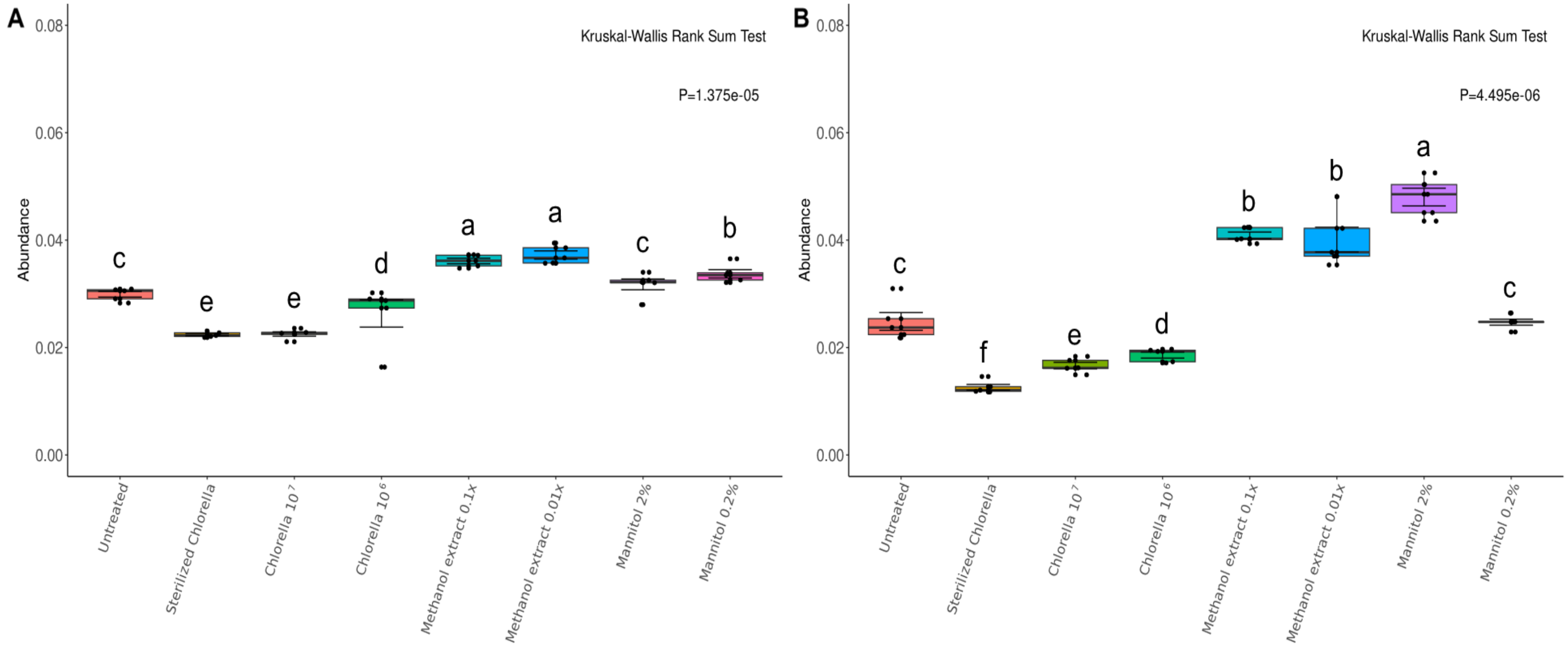

Figure S4. Relative abundance of keystone taxon *Pseudomonas* in each treatment. (A) relative abundance of keystone taxa *Pseudomonas* in each treatment after 12 hr when *Chlorella*, methanol extract, D-mannitol treated to each treatment. (B) relative abundance of the pseudomonas in each treatment after 36 hr. To assess statistical differences, Kruskal-Wallis Rank Sum test was performed, and difference letters represent significantly difference between each group followed by the Conover test (p < 0.05) indicated with the R program (4.3.2).

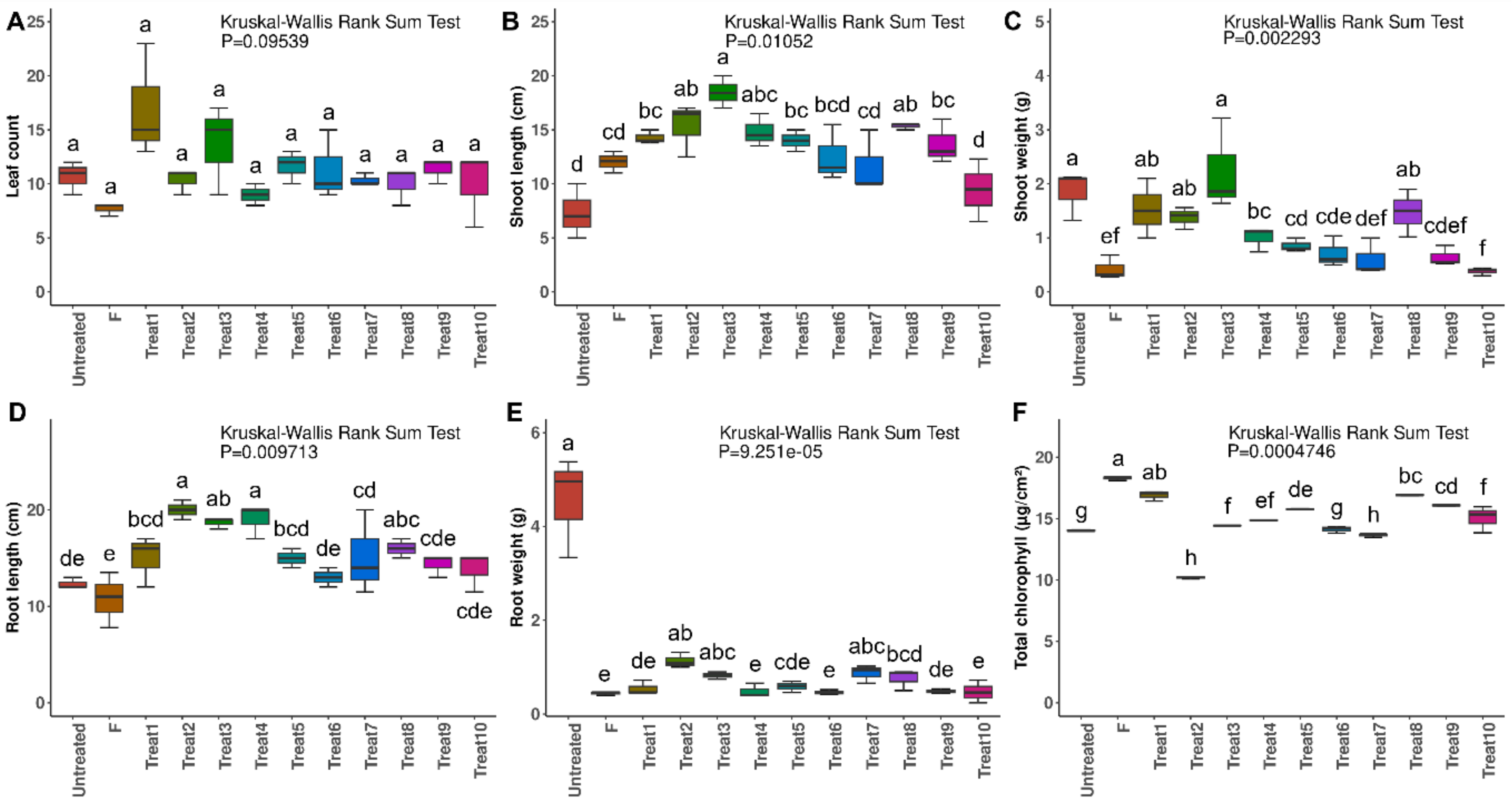

Figure S5. The phenotype of each strawberry treatment treated keystone taxa. Strawberries were grown at 25ºC greenhouse for 3 weeks. The concentrations of keystone taxa [*Pseudomonas* sp. 8C3D12 (8C3D12), *Pseudoduganella rivuli* (FT92W), *Pseudomonas* sp. 6C7F4 (6C7F4), *Brevibacterium renqingii* (REN4)] were 10^6 cfu/mL. *Chlorella fusca* CHK0059 were treated 1/500 dilution of 10^7 cell/mL. The SynCom stocks and CHK0059 stocks were treated 10 mL at three times at strawberries grown for 3 weeks. Growth conditions were conducted in a growth chamber set at a temperature of 28ºC and a humidity of 70%. (A) Difference of leaf counts between each treatment, (B) Shoot length (cm), (C) Shoot weight (g), (D) Root weight (cm), (E) Root

weight (g), (F) Total chlorophyll (μg/cm^2). In x-axis, F: *F. oxysporum* f. sp. *fragariae* F9, Treat1: 8C3D12 + *F. oxysporum* f. sp. *fragariae* F9; Treat2: FT92W + *F. oxysporum* f. sp. *fragariae* F9, Treat3: 6C7F4 + *F. oxysporum* f. sp. *fragariae* F9, Treat4: REN4+ *F. oxysporum* f. sp. *fragariae* F9, Treat5: CHK00059 + *F. oxysporum* f. sp. *fragariae* F9, Treat6: 8C3D12 + FT92W + *F. oxysporum* f. sp. *fragariae* F9, Treat7: 8C3D12 + FT92W + CHK0059 + *F. oxysporum* f. sp. *fragariae* F9, Treat8: SynCom + *F. oxysporum* f. sp. *fragariae* F9, Treat9: SynCom + CHK0059 + *F. oxysporum* f. sp. *fragariae* F9, Treat10: D-mannitol 2% + *F. oxysporum* f. sp. *fragariae* F9. Different alphabets indicate significant differences between treatments according Kruskal-Wallis Rank Sum Test followed by Conover test ($p < 0.05$) using 'bh' method ($p < 0.05$) and indicated with the R program (4.3.2).

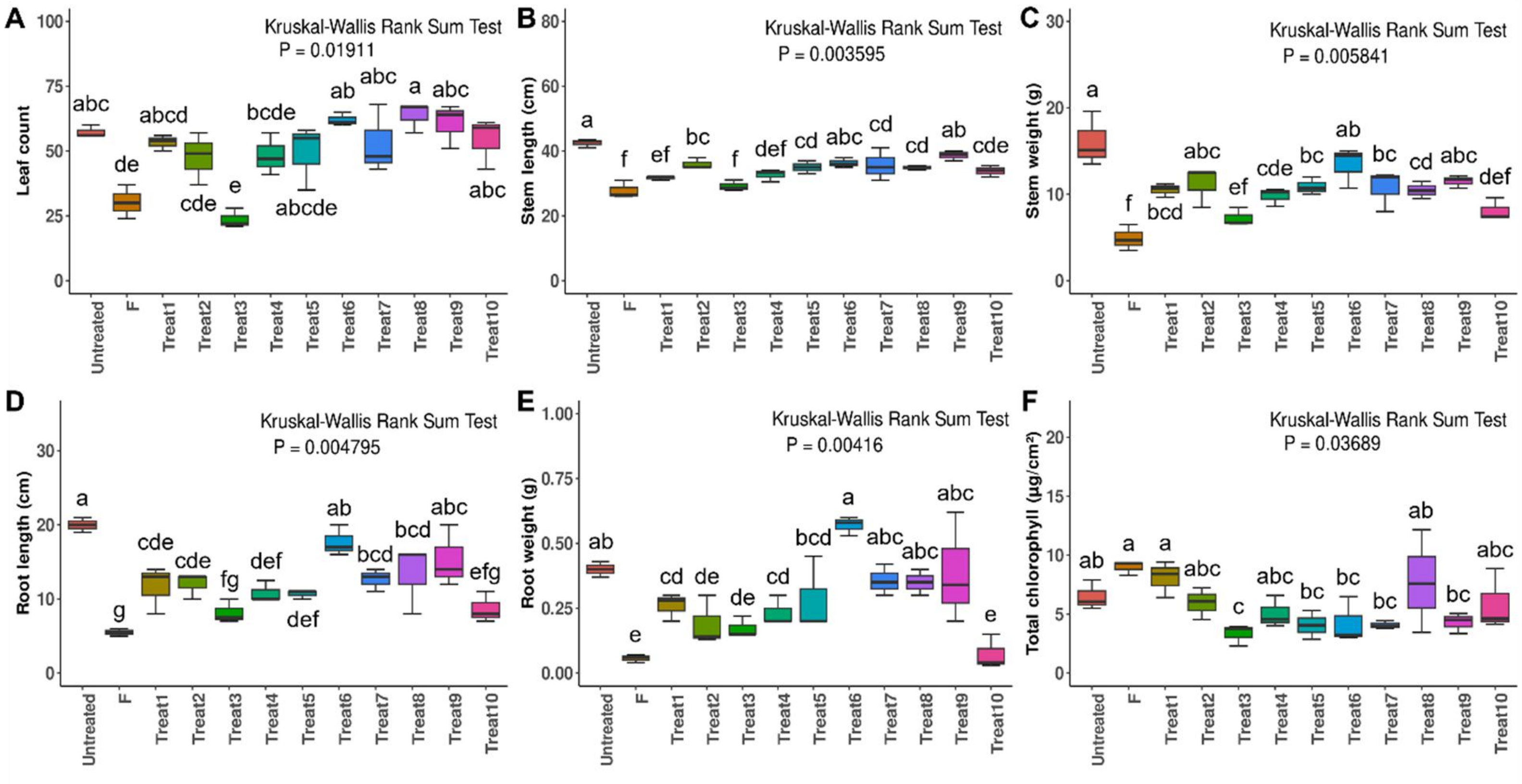

Figure S6. The phenotype of each tomato treatment. Each 10 plants were divided into 3 groups and each index was measured (*n* = 10). After growing until the 4 weeks, the growth degree of each treatment was compared by randomly sampling 3 times for calculating PGP activity of SynCom strains. The tomato growth condition and concentration of treated SynCom (10^6 cfu/mL) strains were the same as that of the SynCom strains treated on strawberries in Figure 7. (A): Difference of leaf counts, (B) Shoot length (cm), (C) Shoot weight (g), (D) Root weight (cm), (E) Root weight (g), (F) Total chlorophyll (μg/cm^2). Treatments were represented by F: *F. oxysporum* f. sp. *lycopersici*, Treat1: 8C3D12 + *F. oxysporum* f. sp. *lycopersici*; Treat2: FT92W + *F. oxysporum* f. sp. *lycopersici*, Treat3: 6C7F4 + *F. oxysporum* f. sp. *lycopersici*, Treat4: REN4+ *F. oxysporum* f. sp. *lycopersici*, Treat5: CHK0059 + *F.*

oxysporum f. sp. *lycopersici,* Treat6: 8C3D12 + FT92W + *F. oxysporum* f. sp. *lycopersici*, Treat7: 8C3D12 + FT92W + CHK0059 + *F. oxysporum* f. sp. *lycopersici*, Treat8: SynCom + *F. oxysporum* f. sp. *lycopersici*, Treat9: SynCom + CHK0059 + *F. oxysporum* f. sp. *lycopersici*, Treat10: Mannitol 2% + *F. oxysporum* f. sp. *lycopersici*. The measured PGP activity values were analyzed for significance using the Kruskal-Wallis Rank Sum Test. As a post-hoc analysis, the PGP activity of each treatment was compared and analyzed using the Conover Test, and the significance between treatments was grouped using the R package (4.3.2).

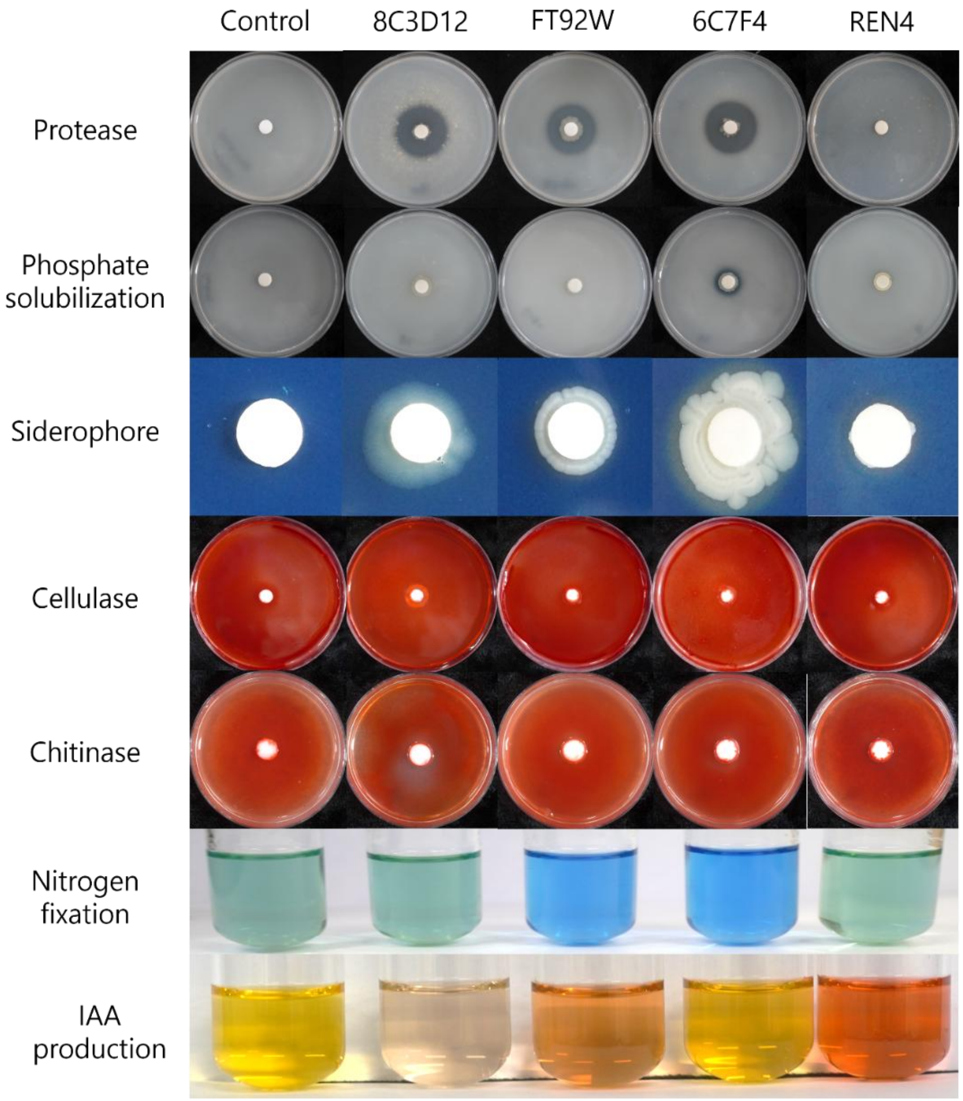

Figure S7. Screening PGP (Plant growth promoting) enzyme activity test of keystone taxa. PGP enzyme test was performed to check the enzymes contained in keystone taxa. The activities of each enzyme were measured by comparison with the control like a clean zone or yellow band around the 8 mm paper disc placed on the plate. The above pictures are enzyme characterization activity from top to bottom, protease, phosphate solubilization, siderophore production, cellulase production, chitinase production, nitrogen fixation and IAA production activity. Protease activity was measured with skimmed milk agar and Phosphate solubilization activity was measured with pikovskaya media. Nitrogen fixation activity was measured with Nfb medium, Cellulase and Chitinase activity were conducted with each media dyed with 1% congo red dye solution. IAA production activity was conducted with Salkowski reagent method.
